# Biomedical Technologies for Androgenic Alopecia Treatment: Advance, Mechanism and Future Direction

**DOI:** 10.34133/research.1312

**Published:** 2026-06-08

**Authors:** Xinyue Cao, Lei Wu, Junkai Wang, Yuanjin Zhao

**Affiliations:** ^1^ Department of Rheumatology and Immunology, Nanjing Drum Tower Hospital, School of Biological Science and Medical Engineering, Southeast University, Nanjing 210096, China.; ^2^ Department of Rheumatology and Immunology, Nanjing Drum Tower Hospital, Clinical College of Nanjing University of Chinese Medicine, Nanjing 210096, China.; ^3^ Co-innovation Center of Neuroregeneration, Nantong University, China.

## Abstract

The most common form of hair loss, androgenetic alopecia (AGA), is characterized by progressive follicular shrinkage. Current research efforts are focused on developing novel therapeutic techniques that exhibit better efficacy and safety profiles. In response to this challenge, biomedical technology has emerged as a multidisciplinary field and offered marked advantages in overcoming skin barriers, enabling controlled drug release, and modulating the perifollicular microenvironment. This review provides a comprehensive overview of advanced biomedical technologies for AGA treatment. We first outline the pathogenesis and introduce some conventional therapies. We then delve into the advantages and practical applications of emerging biomedical technologies, including novel pharmacological formulations, nanotechnology, stem cell-based approaches, and microneedle platforms. Subsequently, our focus shifts to the analysis of current therapeutic strategies in addressing core pathogenic factors through the latest and representative case studies. The review concludes by highlighting major challenges and future directions of these biomedical technology-based approaches, aiming to provide insights for the rational design of next-generation AGA therapies.

## Introduction

The issue of hair loss has become increasingly prominent in modern society, imposing significant physical and psychological burdens on affected individuals [1,2]. The most common type of nonscarring hair loss among the several varieties of alopecia is androgenetic alopecia (AGA), which is mainly defined pathologically by increasing follicular shrinkage and interruption of the hair development cycle [3–7]. Current clinical management of AGA primarily relies on pharmacological therapy, laser therapy, and hair transplantation [8–14]. To date, the U.S. Food and Drug Administration (FDA) has approved only 2 drugs (minoxidil and finasteride) for AGA management [15,16]. Although these agents demonstrate certain therapeutic efficacy, their long-term application is often accompanied by limited outcomes, side effects like contact dermatitis and sexual dysfunction, and poor patient compliance. In contrast, while laser therapy and hair transplantation can improve cosmetic appearance to some extent, the former requires further validation of its long-term efficacy, and the latter is associated with high costs and surgical discomfort. More importantly, neither approach halts the underlying disease progression, and patients frequently require adjunctive long-term pharmacotherapy posttreatment. These limitations underscore the urgent need for the development of novel therapeutic strategies that exhibit increased patient adherence, better safety profiles, and increased efficacy.

In recent years, biomedical technology has emerged as a transformative field integrating materials science, nanotechnology, cell biology, and pharmaceutics [17–22]. Its multidisciplinary nature enables the design of advanced delivery systems capable of precisely targeting pathological mechanisms [23–26]. In the field of AGA treatment, biomedical engineering strategies have demonstrated significant advantages in overcoming the skin barrier, achieving targeted drug release, and perifollicular microenvironment modulation [27,28]. For instance, nanocarriers such as liposomes and nanoparticles have been extensively developed to promote therapeutic agents’ percutaneous penetration [29–32]. Meanwhile, a less intrusive method of penetrating the stratum corneum and delivering bioactives straight to hair follicles (HFs) has already been provided by the microneedle technique [33–35]. Notably, the inherent mechanical stimulation provided by microneedles themselves has also shown unique value in promoting hair growth [36,37]. These technologies not only improve the bioavailability of therapeutic agents but also enable combinatorial interventions simultaneously targeting multiple pathogenic factors, such as inflammation, oxidative stress, and impaired angiogenesis, indicating promising potential in clinical AGA treatment.

**Fig. 1.**
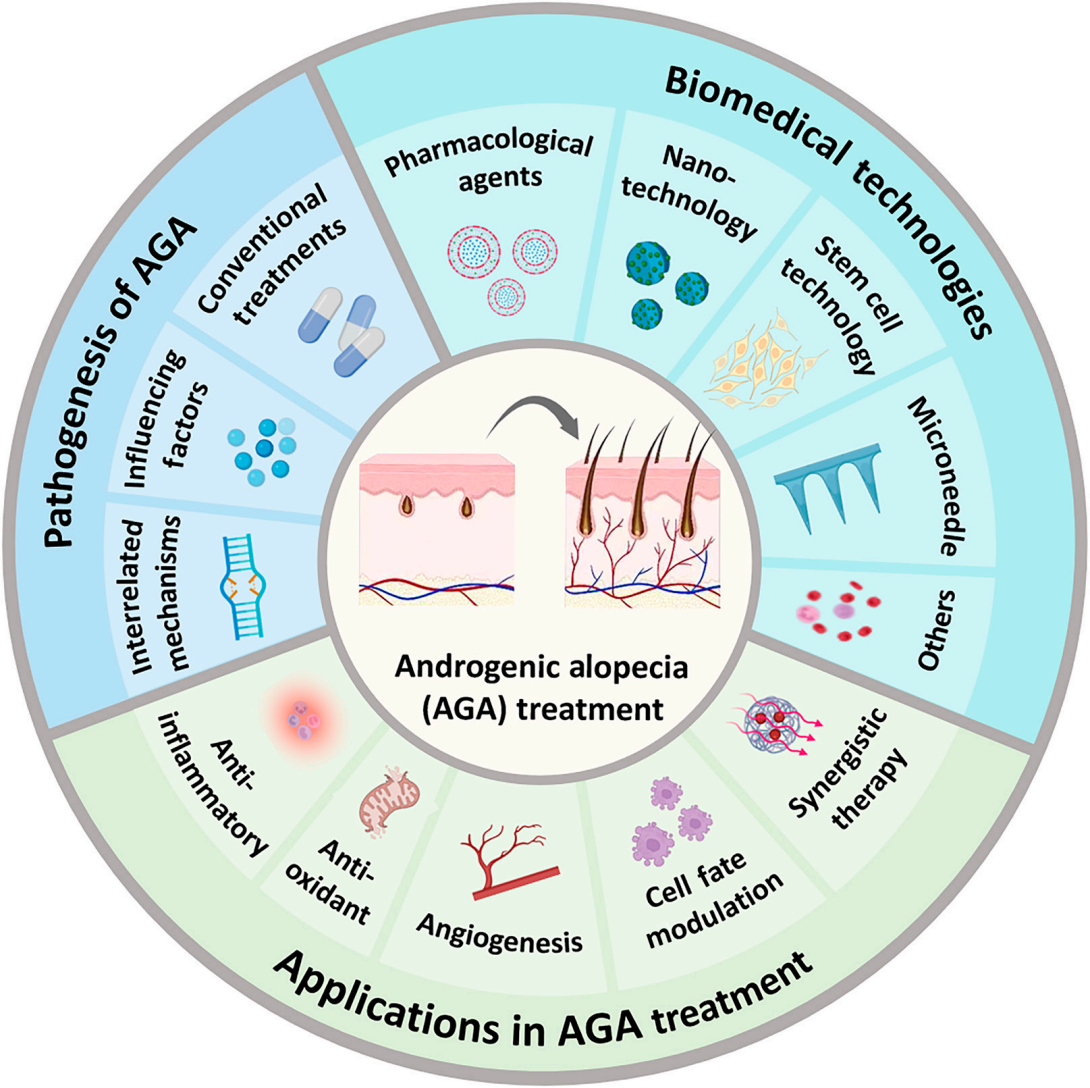
Overview of the advanced biomedical technologies for androgenetic alopecia (AGA) treatment.

This review aims to provide a comprehensive overview of advanced biomedical strategies for the treatment of AGA (Fig. 1). We began by briefly outlining the pathogenesis, influencing factors, and current conventional clinical treatments for AGA, discussing their respective efficacies and limitations. Building upon this foundation, we systematically discussed emerging biomedical engineering technologies with different forms, including novel pharmacological formulations, nanotechnology, stem cell-based therapies, and microneedle platforms, with a focus on their advantages and practical applications in AGA treatment. Subsequently, from the perspective of core pathogenic factors and therapeutic mechanisms in AGA, we used representative case studies to analyze the efficiency of the current therapeutic approaches in addressing anti-inflammatory, antioxidant, proangiogenic, and combination therapy needs. Finally, by synthesizing recent research advancements, we summarized the main obstacles and future paths in this field, aiming to provide novel insights for the rational design of next-generation AGA therapies and to offer research inspirations in this rapidly evolving domain.

## Pathogenesis of AGA

AGA is a prevalent form of nonscarring alopecia. As a chronic condition with a strong hereditary predisposition, its pathological core lies in follicular miniaturization and hair cycle disruption. This section provides an overview of the physiological basis of the hair cycle, followed by a detailed description of the pathological causes of AGA, including the complex interplay of genetic susceptibility, aberrant androgen signaling, local microinflammation, and oxidative stress. Building on this foundation, the mainstay clinical treatment strategies (e.g., pharmacotherapy, laser therapy, and hair transplantation) are introduced, along with a discussion of their respective limitations.

### Disease overview

HFs are mini-organ structures unique to mammals, originating from the neuroectoderm and mesoderm during embryonic development [38–40]. Hair not only performs critical physiological functions such as thermoregulation and mechanical protection but also plays a significant role in individual social interactions and psychological well-being. Consequently, hair disorders often impose considerable psychological distress and social dysfunction on patients [41–43]. Structurally, the HF is composed of epidermal and dermal components. The intricate and precisely regulated interactions between these 2 compartments are fundamental to normal follicular development and cyclical regeneration [40,44]. As one of the few organs in mammals capable of lifelong regeneration, HFs undergo continuous cyclic transformations throughout life, progressing through phases of growth (anagen), regression (catagen), and relative quiescence (telogen) [45]. This cyclic process holds substantial clinical significance, as many hair growth diseases, including AGA and alopecia areata, are pathologically linked to disruptions in the normal hair cycle [7,46,47].

Specifically, during anagen, keratinocytes in the follicular epithelium proliferate rapidly, driving the elongation of the hair shaft. The transition to catagen is marked by the activation of apoptotic signals, leading to the gradual involution of the lower follicular structures. Atrophy of the dermal papilla (DP) and a decrease in the size of the hair matrix, along with a halt in the generation of pigment, are the hallmarks of early catagen. In mid-to-late catagen, the hair matrix progressively diminishes and eventually disappears, while the DP condenses into a compact, spherical structure. Apoptotic cells are also observed in the regressing sebaceous gland. During telogen, the follicle adopts a simplified morphology with a significantly reduced DP, and the fully formed hair shaft transforms into a club hair, which is eventually shed. In the vicinity of the bulge region, which contains hair follicle stem cells (HFSCs), the follicle is currently in a state of relative quiescence. Critical interactions persist between HFSCs and the adjacent dermal papilla cells (DPCs). Upon receiving inductive signals from DPCs during the subsequent cycle, HFSCs become activated, proliferate, and differentiate to generate a new hair structure. Such a process progressively reconstitutes a mature DP and orchestrates the regeneration of a fully functional HF, thereby initiating a new anagen phase [48–51].

In the aspect of nonscarring alopecia, AGA is the most common kind. Pathophysiologically, it does not entail the complete destruction of the HF structure but rather involves aberrant regulation of the hair cycle [52]. AGA is an androgen-dependent and chronic hair loss disease with a strong hereditary predisposition. It typically follows a pattern suggestive of polygenic inheritance, with disease manifestation resulting from complex interactions between susceptibility genes and environmental factors [53]. Its prevalence and phenotypic expression display marked variations across different ethnicities, age groups, and sexes. Clinically, AGA most commonly manifests between the ages of 30 and 50, with a notably higher incidence in males compared to females. Male patients usually have increasing thinning of the vertex (crown) and bitemporal regression of the frontal hairline, while occipital hair is relatively preserved. This is often accompanied by increased scalp sebum production. In female patients, AGA usually manifests as diffuse thinning of hair in the frontoparietal and vertex regions, with the frontal hairline generally remaining intact [43,54,55]. The core pathological features of AGA are follicular miniaturization and hair cycle alteration. This is characterized by a reduced follicles ratio in anagen and an increased ratio in catagen and telogen. Consequently, terminal hairs are progressively transformed into vellus-like hairs, becoming shorter, finer, and lighter in pigmentation, ultimately leading to visible hair thinning and loss. The underlying pathogenesis can be elucidated through the following 3 interrelated mechanisms:

1. Genetic Predisposition and Gene Variants: Numerous genetic loci have been linked to AGA vulnerability by genome-wide association studies. These genes influence the disease phenotype through DNA sequence variants that affect local follicular processes, including oxidative stress responses, inflammatory activity, and epigenetic regulatory mechanisms. For instance, male AGA risk is associated with polymorphism CAG trinucleotide repeat lengths in the androgen receptor (AR) gene [56,57].

2. Heightened AR Signaling in DPCs: DPCs from AGA patients’ balding scalp exhibit elevated expression levels of the AR. Upon stimulation by androgens, particularly dihydrotestosterone (DHT), this overexpressed AR can inhibit the Wnt/β-catenin signaling pathway. Furthermore, complex crosstalk occurs with other signaling cascades, including Shh, Notch, and bone morphogenetic protein pathways. These molecular events collectively trigger the premature entry of HFs into catagen. This pathological signaling may also up-regulate AR coactivators in DPCs, creating a positive feedback loop that further amplifies androgen sensitivity and perpetuates the balding process [43,58–61].

3. Local Production of DHT: Circulating testosterone diffuses via dermal capillaries to reach DPCs. Within these cells, testosterone is irreversibly converted by the enzyme type II 5α-reductase into the more potent androgen, DHT. The DHT–AR complex translocates to the nucleus after binding to the AR, where it alters genes expression essential for controlling the hair cycle. This interference disrupts normal follicular function, leading to premature arrest of anagen, induction of apoptosis in follicular keratinocytes, and the eventual progression of the follicle into telogen, resulting in hair shedding [43,62–64].

### Influencing factors

In addition to the genetic and hormonal factors mentioned above, several other elements play major roles in AGA pathogenesis, including microinflammation, oxidative stress, as well as nutritional status. Microinflammation is a key pathogenic factor in AGA, characterized by a chronic, low-grade inflammatory state around the HFs. This inflammatory environment is primarily driven by lymphocyte infiltration and T-cell activation and is often accompanied by sebaceous gland hyperplasia. These inflammatory cells aggregate in the bulge region where HFSCs are located. By secreting various soluble factors, they directly interfere with stem cell function, causing HF miniaturization and disrupting normal hair growth cycle, and thus establishing it as a core pathological feature of AGA [7,9,65–67]. Furthermore, microcirculatory dysfunction resulting from vascular degeneration in the affected scalp area is a significant contributing factor. Healthy hair growth depends on adequate blood perfusion. In AGA patients, scalp microcirculation is notably impaired. Androgens induce DPCs to release transforming growth factor–β (TGF-β), which encourages vascular calcification and microvascular endothelial cell death, consequently reducing blood flow. This ischemic and hypoxic environment suppresses HFSC activity, forcing HFs to enter the catagen phase prematurely, ultimately resulting in follicular atrophy and miniaturization [7,9,68,69].

Oxidative stress is another emerging candidate in AGA pathogenesis. Mitochondrial dysfunction disrupts the energy metabolism of HF cells by reducing adenosine triphosphate production and increasing reactive oxygen species (ROS). Excess ROS accelerates follicular degeneration by oxidatively damaging cellular components and initiating apoptotic pathways. The shape, motility, proliferation, senescence, and TGF-β signaling of DPCs can all be markedly changed by environmental ROS portion. Due to their markedly increased sensitivity to oxidative stress, DPCs from balding scalps would release more TGF-β (a negative regulator of hair development) in response [70–72]. Concurrently, nutritional status directly affects the hair cycle and structural integrity. Deficiencies in vitamins (e.g., vitamins B12, D, and E, and folate), trace elements (e.g., magnesium, calcium, zinc, copper, selenium, and iron), or amino acids (e.g., histidine, leucine, and alanine) can disrupt the normal hair cycle and compromise hair shaft structure and pigmentation [9,73–75]. Moreover, significant fibrotic changes are observed around the HFs in approximately 37% of AGA patients. This fibrosis is closely linked to chronic inflammation induced by prolonged, involuntary tension in the scalp muscles. Fibrotic changes compress blood vessels, further exacerbating local microcirculatory disturbances and tissue hypoxia, thus creating a vicious cycle. Additionally, androgens can promote fibrosis by stimulating collagen synthesis and up-regulating TGF-β1 expression, which directly damages HFSC function and accelerates HF miniaturization and hair loss progression [7,9,76,77].

To sum up, as research into AGA pathogenesis develops, therapeutic strategies focused on modulating HF microenvironment and supplementing key nutrients are emerging as potential approaches to slow the progression of hair loss.

### Conventional treatments

**Fig. 2.**
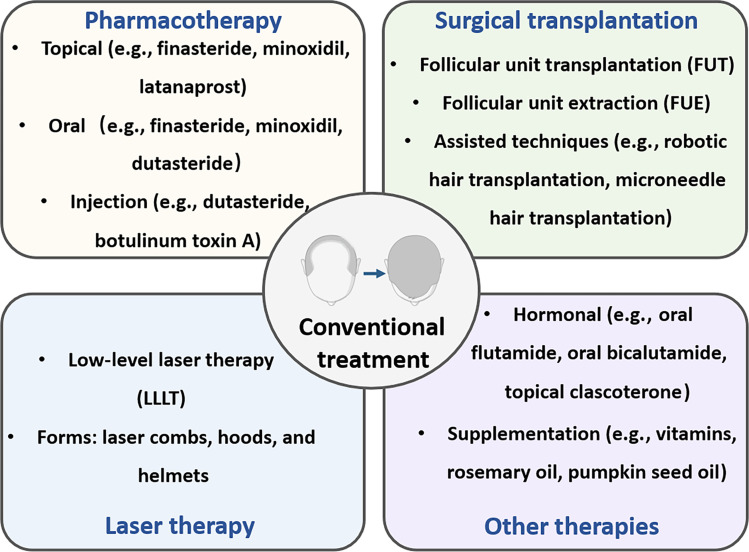
Overview of the conventional androgenetic alopecia (AGA) treatments.

Currently, the primary clinical approaches for treating AGA include pharmacotherapy, laser therapy, and hair transplantation, as schemed in Fig. 2. Regarding pharmacotherapy, minoxidil and finasteride are the only 2 medications currently approved by the U.S. FDA to treat AGA. Oral and topical administration are the most commonly used routes of drug delivery [12,78]. Minoxidil is a potassium channel opener that mainly increases perifollicular blood flow and dilates vascular smooth muscle to stimulate hair growth. However, its effect is mainly manifested in increasing hair diameter and weight, with a relatively weak effect on increasing hair count. It requires long-term use and is prone to adverse effects like contact dermatitis, pruritus, as well as facial hypertrichosis [79–81]. Finasteride, a competitive inhibitor of type II 5α-reductase, blocks DHT production in the serum and scalp. However, its long-term use may lead to risks such as gynecomastia, decreased libido, anxiety, depression, and insulin resistance. It is contraindicated in female patients [82–84]. Notably, with advancing research, novel therapeutic targets are under development, including AR antagonists, prostaglandin analogs, drugs targeting the Wnt signaling pathway, and Janus kinase inhibitors [85–90]. However, the efficacy and safety of these agents still require substantial evidence-based validation.

Low-level laser therapy (LLLT) is a more recent technique for treating various types of hair loss disorders, with products available in forms such as laser combs, hoods, and helmets. Clinical studies have demonstrated that following LLLT treatment, both male and female patients with AGA show significant increases in hair count, density, thickness, and tensile strength. Although the precise mechanism of action remains incompletely understood, LLLT is thought to have the ability to stimulate hair growth by up-regulating the expression of growth factors, activating dormant HFs, and increasing local blood flow. However, this treatment modality can also lead to adverse effects in some patients, including scalp pruritus, erythema, follicular damage, and scarring. Furthermore, the long-term efficacy of LLLT requires further evaluation [91–94].

In contrast, hair transplantation surgery is a currently popular method for treating AGA, offering the advantage of selective adjustment of the transplant area. However, the quality of evidence supporting its efficacy varies considerably. Factors such as differing surgical techniques, varying methods of hair graft harvesting, thinning of the donor site scalp in patients with severe AGA, and insufficient donor hair area can contribute to a low survival rate of transplanted hair grafts. Importantly, this surgical approach fails to halt the progression of AGA itself, while patients often require extra adjuvant pharmacotherapy. Additionally, the surgical procedure itself carries potential complications, including infection and bleeding. Therefore, hair transplantation should be regarded as a comprehensive treatment option that requires strict adherence to indications and combination with medical therapy, rather than a standalone, permanent solution [95–97].

## Frontiers of Biomedical Technologies

Biomedical technology, as a cutting-edge field integrating materials science, nanotechnology, and regenerative medicine, has brought significant advances to the treatment of AGA. Its core advantages lie in effectively addressing the limitations of conventional therapies through precise modulation of drug delivery, overcoming the skin barrier, and regulating the HF microenvironment. This section systematically elaborates on the representative technologies within this domain, including the refinement of pharmaceutical formulations based on novel delivery systems, advanced regenerative strategies employing nanotechnology and stem cell technology, as well as the development of microneedle platforms with physical penetration-enhancing functions, etc. Of note, in current laboratory studies on AGA, the commonly used animal model is the C57BL/6 mouse, and the modeling method primarily involves topical application or subcutaneous injection of testosterone or DHT on the dorsal skin to simulate androgen-induced follicular miniaturization.

### Novel pharmacological formulations

As mentioned above, in the field of AGA treatment, commonly used medications that control blood vessels and encourage hair growth are minoxidil and finasteride. Current research in the field of drug delivery is concentrated on creating innovative drug delivery systems to improve delivery effectiveness, accomplish precise and controlled drug release, and permit prolonged release effects. To meet this demand, the glycosidic compound stevioside was utilized as a novel solubilizing agent to incorporate minoxidil. Stevioside self-assembles in aqueous solution to form a core–shell structure featuring a hydrophobic cavity, which encapsulates the poorly soluble drug minoxidil through intermolecular interactions, eliminating the need for complex preparation processes. The resulting complex achieves controlled and long-acting delivery of minoxidil, significantly enhancing its solubility and skin permeability [98]. Besides, Xu’s team [99] developed a novel in situ thermosensitive hydrogel (named as ICPG) to realize topical codelivery of minoxidil and finasteride for AGA therapy. Here, an ionic liquid (IL) was first synthesized using choline and salicylic acid, which markedly increased the solubility of both drugs. This IL was then formulated into a hydrogel using poloxamers and 2-hydroxypropyl-β-cyclodextrin. The resulting ICPG exhibited thermosensitive properties, transitioning from solution to gel at 32 °C. In skin studies, ICPG disrupted the stratum corneum barrier, significantly enhancing targeted delivery and accumulation of the 2 medicines to HFs. This therapeutic efficacy has been further evaluated in AGA mouse models, revealing a significant increase in both the hair coverage area and the hair density. Through histological, immunohistochemical, and immunofluorescence analyses, the drug-loaded ICPG demonstrated superior hair regrowth. Reverse transcription polymerase chain reaction analysis further elucidated the underlying mechanism, confirming the formulation’s potential as a promising, solvent-free strategy for AGA treatment.

Similarly, Zhang’s team [100] has developed a novel IL-based system to deliver minoxidil and epigallocatechin gallate for treating AGA. A multifunctional choline and geranic acid IL (CGIL) was synthesized through a 1-step approach. CGIL effectively solubilized minoxidil, achieving high drug loading while also possessing inherent hair growth-promoting properties. The formulation (ME@CGIL) was designed to overcome the stratum corneum barrier, prolong skin drug retention, and provide a dual therapeutic mechanism: Epigallocatechin gallate eliminated excessive ROS to reduce oxidative stress, while minoxidil promoted angiogenesis by up-regulating the vascular endothelial growth factor (VEGF). Even at lower doses and less frequent administrations, ME@CGIL therapy accelerated the transition of HFs from the telogen to the anagen phase in an AGA mouse model when compared to commercial minoxidil solution. The ME@CGIL group demonstrated better hair coverage and regenerated hair quality, according to histological examination. Thus, it is believed that this IL-based codelivery system presents a promising and efficient strategy for AGA treatment.

In addition to therapies involving these 2 FDA-approved drugs, it is worth noting that traditional Chinese medicine strategies have also been applied in the treatment of AGA. These primarily include the use of herbal medicines such as *Polygonum multiflorum*, Rehmanniae Radix Praeparata, turmeric, and quercetin (Que), as well as physical therapies like plum-blossom needle therapy and scraping therapy (gua sha) [101,102]. In a recent study, a nanosystem (PDA@QLipo) incorporating polydopamine (PDA) and Que within a liposomal carrier was fabricated to remodel the perifollicular microenvironment for treating AGA [103]. PDA was selected for its antioxidant properties and ability to supply exogenous melanin, while Que was chosen to promote angiogenesis by activating the mitogen-activated protein kinase/cyclic adenosine monophosphate response element–binding protein signaling pathway. A liposome formulation was employed to encapsulate these agents, enhancing scalp affinity and enabling effective transdermal delivery facilitated by a simple rolling administration technique. The therapeutic efficacy was evaluated in an AGA mouse model. PDA@QLipo effectively scavenged ROS, increased melanin content, and promoted neovascularization in the HF vicinity. Compared to the FDA-approved drug minoxidil, treatment with PDA@QLipo resulted in superior hair regrowth, as evidenced by significantly higher new hair density and increased hair diameter. Through simultaneously alleviating oxidative stress and promoting angiogenesis, this dual-action nanosystem effectively remodeled a favorable microenvironment for DPCs, presenting a promising and synergistic strategy for clinical AGA therapy.

**Fig. 3.**
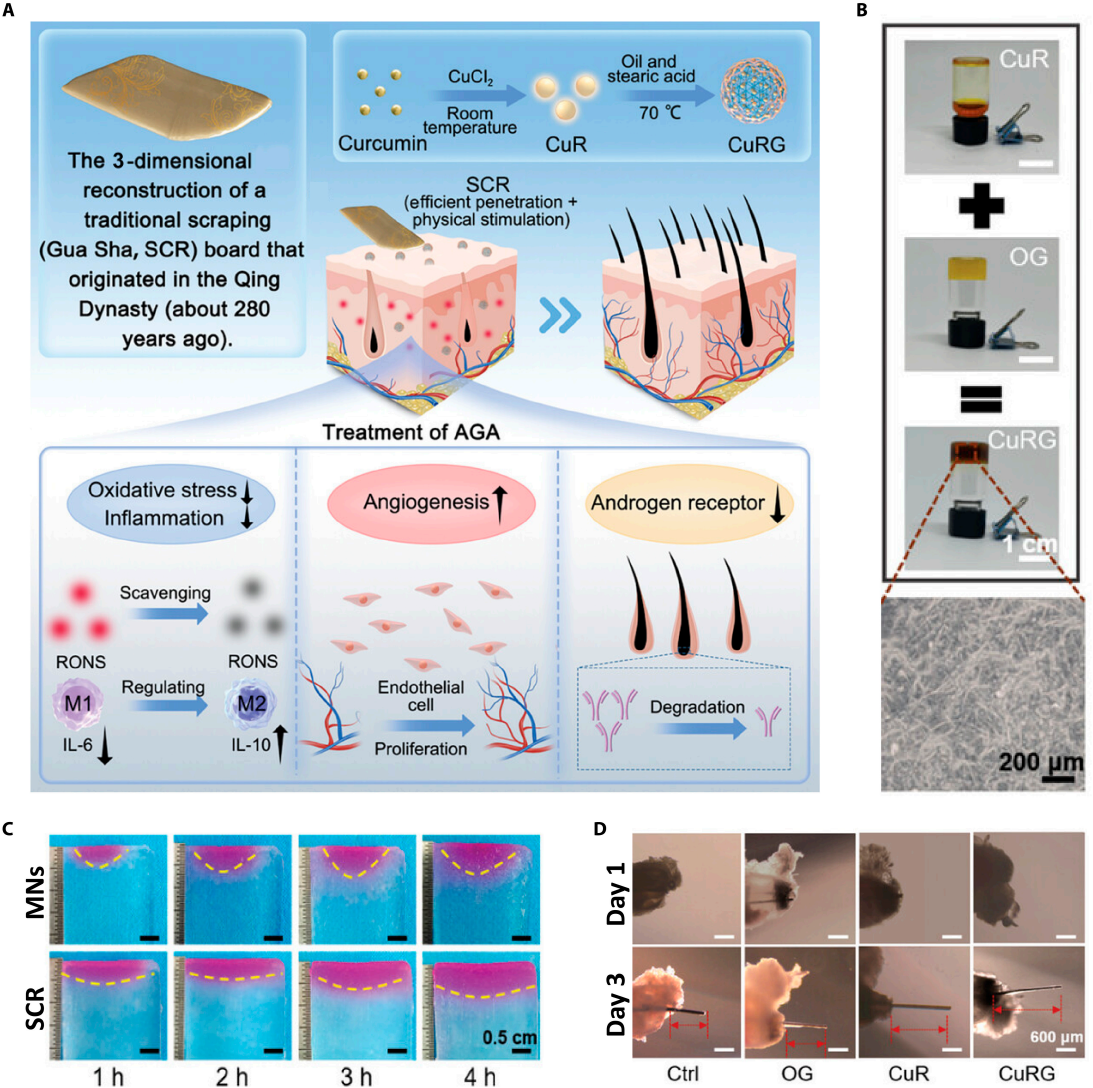
(A) The schematic diagram of CuRG combined with the scraping drug delivery system in androgenetic alopecia (AGA) therapy. Here, RONS is reactive oxygen and nitrogen species. (B) The preparation process and microscope image of CuRG. (C) Optical images of RhB permeation in agar after different treatments. (D) Representative micrographs of hair follicle (HF) tissue after various treatments in vitro [104]. Copyright 2024, Wiley-VCH GmbH.

It is notable that Wang et al. [104] have applied the Chinese scraping strategy to AGA treatment. They suggested a copper–curcumin oleogel (CuRG)-based and scraping-enhanced transdermal administration device for the multipurpose treatment of AGA, as schemed in Fig. 3A. Here, a straightforward technique was used to create copper–curcumin (CuR) coordination nanoparticles, which showed strong reactive oxygen and nitrogen species scavenging capability, high stability, and biocompatibility. CuR was added to an oleogel (OG) base made of stearic acid, soybean oil, and ginger essential oil to produce CuRG (Fig. 3B). The oleogel components, particularly the unsaturated fatty acids, enhanced skin permeability, while the ginger oil contributed additional antioxidant properties. To improve dermal delivery, a traditional scraping technique was employed, as evidenced in Fig. 3C. This physical stimulation not only facilitated deeper penetration of CuR to the epidermis and dermis but also provided mechanical stretching that independently activated hair stem cells (Fig. 3D). Upon delivery, the system released curcumin and copper ions to simultaneously intervene in multiple pathological pathways: altering the perifollicular milieu through the degradation of ARs, the reduction of oxidative stress, the promotion of vascularization (by Cu^2+^-induced VEGF overexpression), and the control of inflammatory responses. The synergistic therapeutic efficacy was evaluated in an AGA model and compared against minoxidil, demonstrating the feasibility of this multifunctional approach for synergistic AGA therapy.

### Nanotechnology

**Fig. 4.**
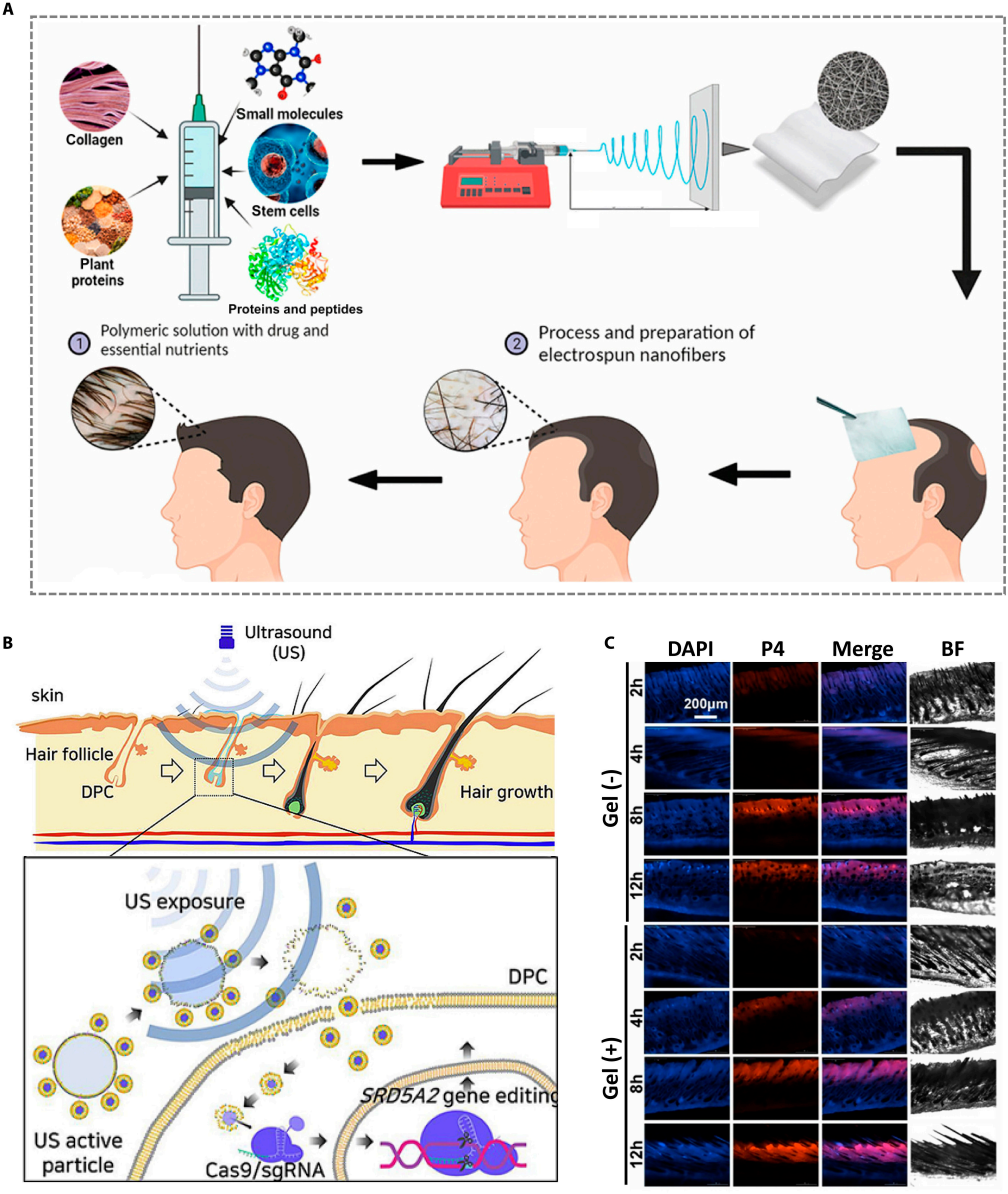
The schematic diagram of the complete electrospinning process and the application of nanofiber patch on the scalp [110]. Copyright 2023, Elsevier B.V.

In the field of AGA treatment, nanotechnology is primarily categorized into nanocarrier systems and nanodrug systems. Nanocarrier systems are used to deliver active ingredients, whereas nanodrug systems themselves possess therapeutic functions as active components [105–107]. In recent years, electrospun nanofibers within nanocarrier systems have attracted significant attention due to their high porosity, ideal hygroscopicity, and superior oxygen exchange rate. They are fabricated mainly via electrospinning, a straightforward and practical technique that applies an electric potential to a polymer solution to generate fibers with nanoscale diameters [108–110]. In the design of patches contact with the scalp, the selection of polymers is crucial. Nanofiber polymers can be classified into 3 categories: synthetic polymers, including polylactic acid, polyglycolic acid, polyvinyl alcohol, and polycaprolactone; natural polymers, such as collagen, fibroin, hyaluronic acid (HA), chitosan, alginate and their derivatives; and hybrid polymers, for instance, blends of polycaprolactone and collagen [110–114]. Through diverse combinations and drug loading, these polymers can effectively deliver active ingredients, thereby enhancing the therapeutic efficacy for AGA, as schemed in Fig. 4 [110]. For example, a technique for creating electrospun gelatin nanofibers with silver nanoparticles was presented by Tura et al., and it successfully promoted the formation of rabbit HFs [115]. In another work, stable polyvinyl alcohol-based nanofibers were fabricated for the delivery of minoxidil sulfate, achieving high encapsulation efficiency and sustained release while protecting the drug from degradation, thereby offering an ideal therapeutic effect [116].

In addition to nanofibers, the liposome also plays an important role in nanocarrier systems [117–120]. Recently, Yoon’s team [121] used an ultrasound-activated microbubble-conjugated nanoliposome system for topical delivery of CRISPR-Cas9 protein complexes, which could target the SRD5A2 gene in DPCs. The system was designed to deliver Cas9 nuclease–single-guide RNA complex directly as a protein-based formulation, offering better gene editing efficiency with reduced toxicity and off-target outcomes compared to viral or plasmid vectors. To overcome challenges of protein degradation and poor skin penetration, the Cas9/single-guide RNA complexes were encapsulated within nanoliposomes, which were then conjugated to microbubbles. Upon topical application, high-frequency ultrasound was applied to activate the microbubbles. The resulting cavitation produced sonoporation, temporarily disrupting the stratum corneum barrier and enhancing local transport of the gene editing complexes to DPCs in the HF. This nonviral approach enabled site-specific delivery of the Cas9 machinery to edit SRD5A2, the enzyme responsible for converting testosterone to DHT, potentially expanding the therapeutic platform for targeted genome editing in hair loss diseases.

Furthermore, liposomes have been used to encapsulate cardamonin (CAR@Lip), which was further incorporated into a gel matrix (CAR@Lip Gel) for enhanced topical treatment of AGA. The HF pathway is a vital route and drug reservoir for topical distribution, and the CAR-loaded liposomes were created to overcome CAR’s low bioavailability and poor water solubility. The goal of the liposomal gel formulation was to increase drug penetration through the follicular sebaceous gland route and the stratum corneum, while simultaneously enhancing physical stability and patient compliance. In order to assess the effectiveness of skin administration, the researchers measured drug dispersion in the skin and examined nanoparticle movement, paying particular attention to the “ratchet effect” on HFs that promotes drug accumulation. Notably, the antiandrogen effects of the nanoformulations were examined in both in vitro and in vivo tests. Additionally, the study elucidated CAR’s mechanism of action against AGA, providing a new natural plant-based strategy for topical AGA treatment [122].

As for nanoparticles, in a typical work, minoxidil-loaded polymeric nanoparticles were formulated using natural polymers chitosan (CS) and HA via the ionic gelation method. CS was selected for its ability to provide sustained drug release, enhance stratum corneum penetration, and biodegradability, while HA acted as a crosslinking anion and permeation enhancer due to its hydration properties, also contributing to hair growth by promoting keratinocyte functions. The prepared minoxidil-loaded polymeric nanoparticles were subsequently incorporated into a hydrogel to create a topical nanohydrogel formulation. This system was designed to prolong drug contact time at the application site, reduce side effects associated with conventional minoxidil formulations, and enhance permeation across the stratum corneum [123]. Moreover, Mohammadi and colleagues [124] synthesized methylated aminobenzyl carboxymethyl chitosan (MCS) and minoxidil-loaded chitosan (CS) nanoparticles. The 2 types of nanoparticles were tested for cytotoxicity, skin penetration, drug release, physicochemical characteristics, and in vivo hair growth. The findings showed that whereas CS nanoparticles demonstrated higher epidermal penetration, MCS nanoparticles displayed a longer sustained-release profile. Notably, compared to the commercial formulation, both nanosystems demonstrated superior efficacy in encouraging hair growth in the treatment of AGA.

**Fig. 5.**
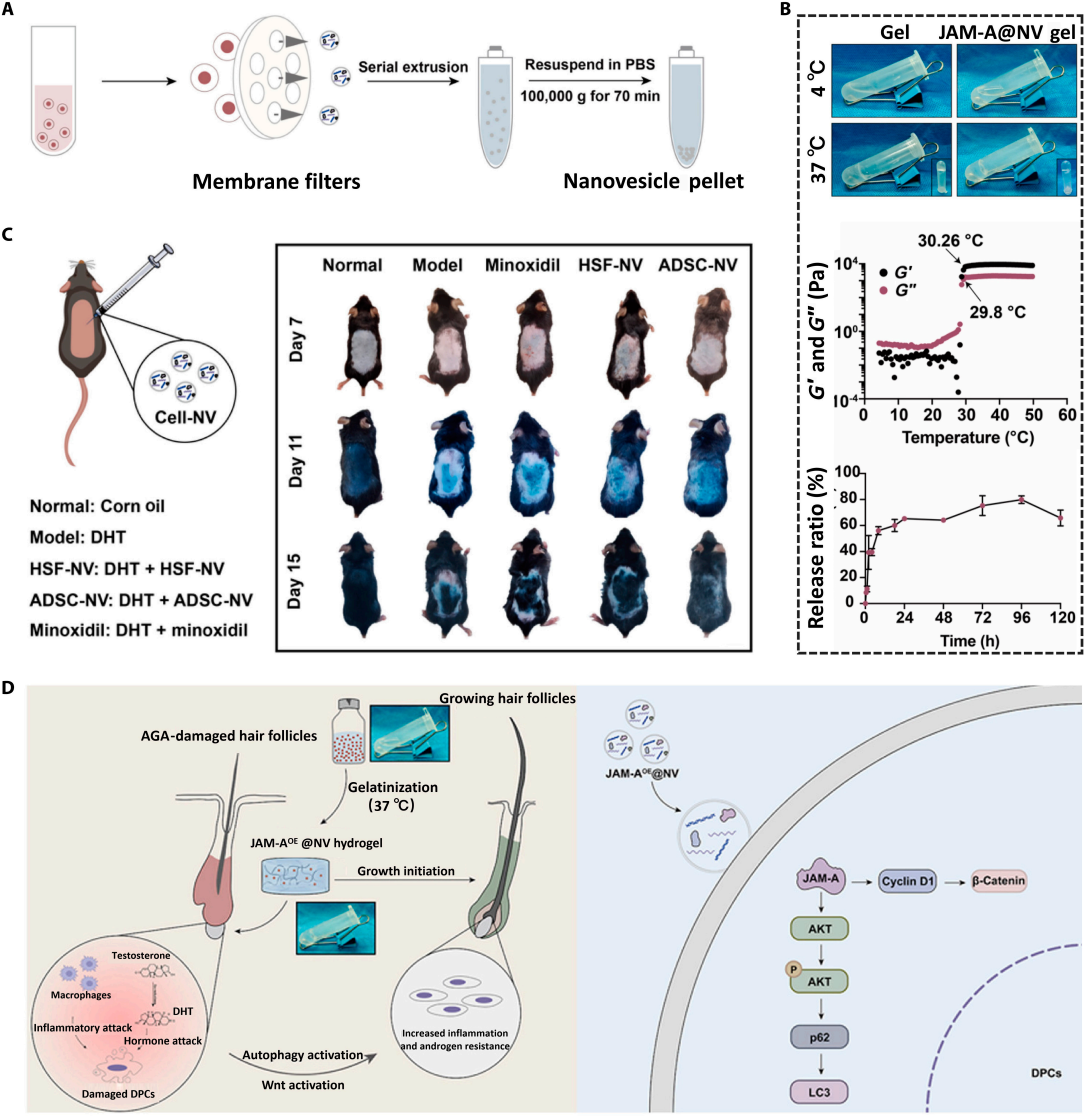
(A) Schematic of the preparation of the adipose-derived stem cell nanovesicles (ADSC-NVs). (B) Temperature response behavior of JAM-A@NV Gel. (C) Effect of ADSC-NVs on hair regeneration in an androgenetic alopecia (AGA) mouse model. (D) Schematic diagram of the mechanism of JAM-A^OE^@NV Gel promoting AGA hair regeneration [129]. Copyright 2024, the author (s).

On the other hand, the nanodrugs’ intrinsic tiny size and special physicochemical characteristics (such as high specific surface area, adjustable surface charge, and superior biocompatibility) allow them to successfully pass through the skin’s stratum corneum barrier. They penetrate deeply into the epidermis and dermis via transcellular or paracellular pathways, enabling precise delivery to the pathological regions of HFs [125–128]. For instance, cell-derived nanovesicles, produced from mechanical extrusion, have provided a cost-effective and biomimetic alternative to exosomes for therapeutic applications. Therefore, researchers developed engineered adipose-derived stem cell (ADSC) nanovesicles (ADSC-NVs) overexpressing junctional adhesion molecule A (JAM-A) and incorporated them into a thermosensitive poloxamer 407 hydrogel for sustained topical delivery to treat AGA (Fig. 5A and B) [129]. Here, ADSC-NVs were initially prepared via mechanical extrusion and demonstrated superior hair regrowth efficacy (larger hair coverage area with faster pigment deposition) compared to human skin fibroblasts nanovesicles, as shown in Fig. 5C. Mechanistic investigations showed that JAM-A protein, which was transported by ADSC-NVs, was important because it encouraged autophagy in DPCs, strengthening their defense against inflammatory and DHT-induced damage. Bioinspired artificial nanovesicles with overexpressed JAM-A were created in order to enhance this therapeutic effect. They were then combined into a thermosensitive hydrogel to produce a sustained-release platform that allows for the effective and prolonged distribution of the restorative JAM-A protein to HFs (Fig. 5D). This cell-free strategy leverages engineered nanovesicles to target a newly identified mechanism, offering an advanced and promising approach for hair regeneration in AGA.

### Stem cell-based technology

As an emerging treatment for alopecia, stem cell-based therapies—which primarily include stem cell transplantation, transplantation of conditioned media obtained from stem cells, and transplantation of exosomes derived from stem cells—have attracted a lot of attention. The process of hair transplantation is basically a clinical use of stem cell treatment. Researchers have attempted to isolate and culture cells from the dermis, epidermis, or other sources using various experimental methods, aiming to generate bioengineered HFs through heterotopic or intradermal transplantation that are capable of producing hair and recapitulating the physiological function and cyclic regeneration of normal HFs [130–134]. For example, Nilforoushzadeh et al. [135] evaluated the regenerative potential of cultured mature DPCs to induce HF growth when injected into the skin of nude mice. Initially, the cultured DPCs were observed to multiply with CD200 expression, forming fusiform colonies within 3 to 5 d. Following the third passage, they formed an extracellular matrix. Histopathological examination of mice injected with 1.2 × 10^6^ DPCs revealed that HF-like structures had developed at the injection areas. In a related research, Ibrahim et al. [136] treated AGA and refractory patchy alopecia using autologous bone marrow mononuclear cells, including stem cells, and compared the results with those of autologous HFSCs. A single intradermal injection of 1 ml containing 100,000 cells/cm^2^ via a 26-gauge needle led to significant improvement across all treated groups. Notably, the therapeutic effect was similar regardless of the cell source. As for the reason for their effectiveness, the cellular diversity of bone marrow mononuclear cells includes progenitor cells, hematopoietic stem cells, mesenchymal stem cells, and inflammatory cells. They also possess the capacity to differentiate into different cell types and secrete bioactive molecules like VEGF, which stimulates angiogenesis, immunomodulatory, and anti-inflammatory factors that have antiapoptotic effects.

Furthermore, ADSCs, derived from the perivascular niche, not only activate HFSCs and promote vessel formation through releasing growth factors such as VEGF, hepatocyte growth factor, platelet-derived growth factor, and insulin-like growth factor 1 (IGF-I) but also maintain the homeostasis of the HF microenvironment through their immunomodulatory properties, thus positioning them as an ideal cell population for treating AGA [134,137–139]. Current therapeutic strategies typically utilize their paracrine factors and derived exosomes. In clinical trials, Park et al. [140] reported that ADSC-derived exosomes combined with microneedling significantly enhanced hair density and thickness in AGA patients without adverse reactions, suggesting ADSC exosomes as an effective treatment for hair loss. In addition, experimental findings revealed that ADSC exosomes inhibit the TGF-β1/SMAD2 pathway via miR-574-3p and miR-125a-5p, thereby reversing DHT-induced damage to DPCs. Knockdown of either microRNA (miRNA) alone cannot affect the therapeutic outcomes, whereas simultaneous knockdown of both significantly reduced it, indicating that these 2 miRNAs play an important role in exosome-based AGA therapies [141].

### Microneedles

Microneedle arrays typically consist of several microneedles with lengths less than 1,000 μm and are mainly sorted into solid, hollow, coated, dissolvable, and hydrogel types, capable of loading both hydrophilic and hydrophobic therapeutic agents [142–144]. By virtue of their physical length to penetrate the stratum corneum, transdermal administration via microneedles can effectively circumvent issues such as gastric irritation, hepatic first-pass effect, and poor patient compliance associated with oral administration. Furthermore, the minimally invasive mechanical stimulation caused by microneedles can itself activate skin repair mechanisms, promoting local blood flow and key growth factor secretion thereby contributing to HF activation [145,146]. Specifically, to deliver traditional Chinese medicine extracts via microneedle platform, Hong et al. [147] introduced a double-network cross-linked hyaluronic acid methacrylate/HA hydrogel as microneedles containing *Platycladus orientalis* leaf extract (PO-ex). It is notable that, by eliminating ROS, PO-ex could successfully enhance the conditions for the growth of HF cells. This design ensures sufficient mechanical strength and controlled release of PO-ex. Upon skin penetration, HA, as an extracellular matrix component, also helps modulate the HF microenvironment to promote regeneration. Besides, Wang’s team [148] developed double-layer microneedles loaded with polygonum for AGA treatment. The needle tip offers enough strength for skin penetration, while the flexible base ensures better skin adhesion. Animal experiments confirmed that polygonum microneedles promote HF growth, offering a promising strategy for AGA treatment.

**Fig. 6.**
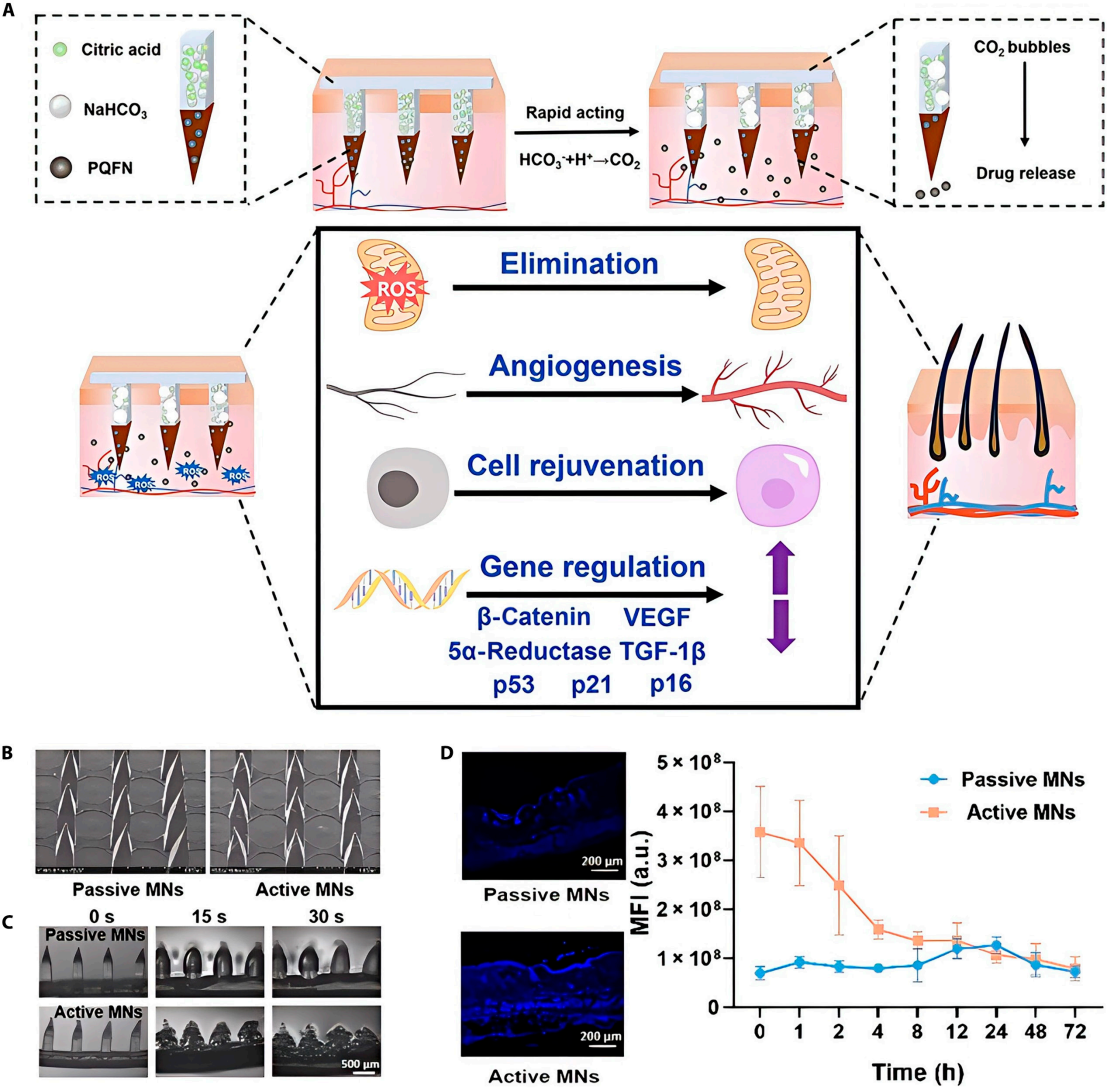
(A) Schematic illustration of the Quercetin + Puerarin + metal ion nanoparticle (PQFN)-loaded gas-propelled microneedles for enhanced topical treatment of androgenetic alopecia (AGA). (B) The scanning electron microscopy (SEM) images of passive microneedles and active microneedles. (C) The gas production capacity of passive microneedles and active microneedles under aqueous environment. (D) Fluorescence images and intensity of frozen tissue sections of living mouse skin after insertion by the passive microneedles and active microneedles [149]. Copyright 2025, Elsevier B.V.

**Fig. 7.**
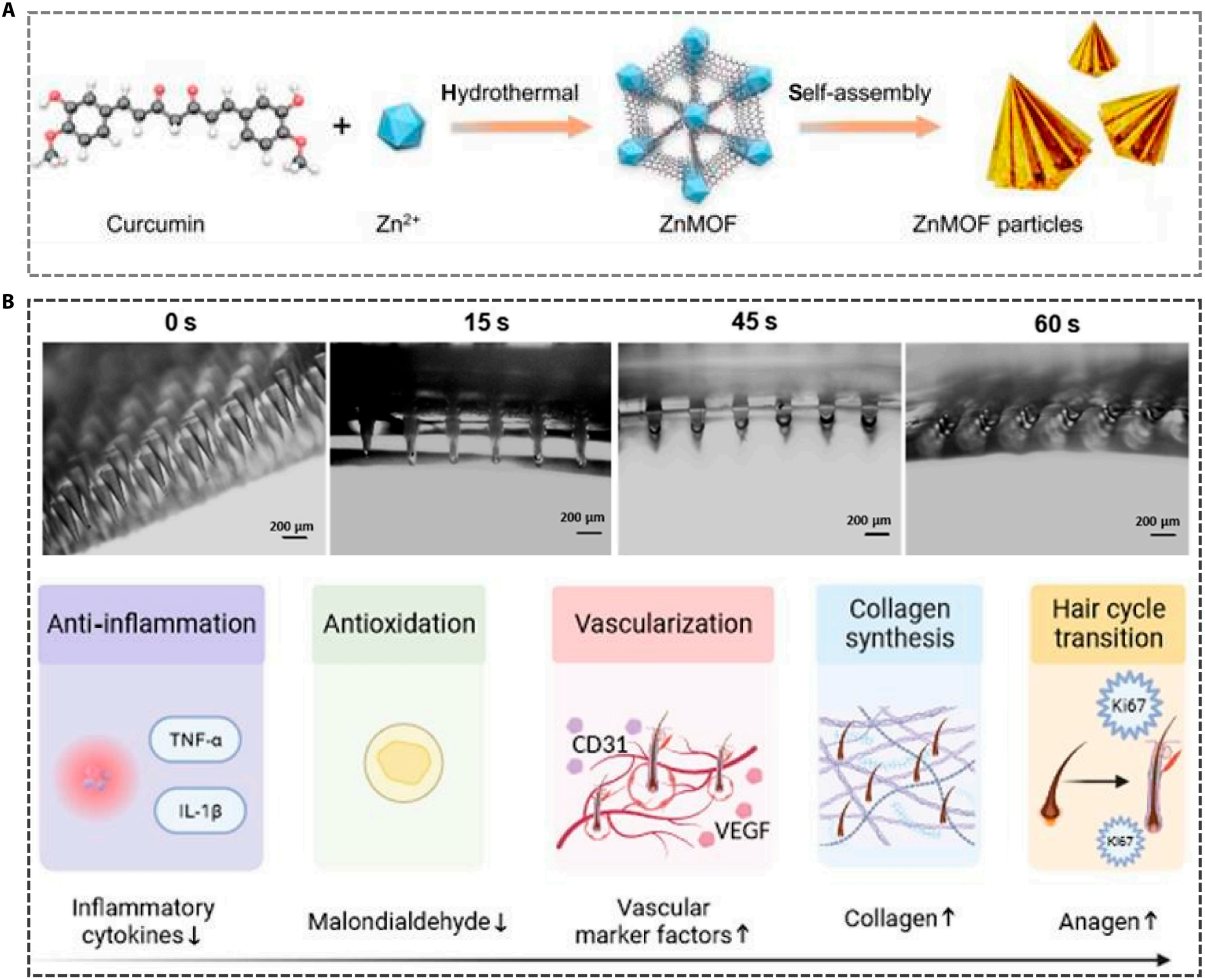
(A) Schematic illustration of the synthesis of curcumin–zinc framework (ZnMOF) microsphere [150]. Copyright 2023, the author (s). (B) Dissolution of microneedles at different times of insertion into the skin. The schematic illustration of the underlying therapeutic mechanism [154]. Copyright 2023, Elsevier B.V.

Notably, Que (a flavonoid) and Puerarin (Pue, an isoflavone) have been proven to exhibit antioxidant, antiaging, and vasodilatory properties. However, their poor water solubility limits clinical application in AGA. To address this, Peng et al. [149] synthesized Quercetin + Puerarin + metal ion nanoparticles (PQFNs) by complexing Que and Pue with metal ions, leveraging metal–polyphenol coordination to enhance solubility, loading capacity, and enzyme-like catalytic activity (Fig. 6). These nanoparticles were integrated into gas-propelled microneedles containing effervescent agents. In order to overcome the passive diffusion restrictions of traditional microneedles, the effervescent agents react with the interstitial fluid upon skin insertion, producing gas bubbles that produce a local vortex field. This actively propels PQFN release and deep penetration into HFs. Microneedles themselves pierce the stratum corneum minimally invasively, enhancing drug delivery and patient compliance, while mechanical stimulation accelerates hair regrowth via Wnt/β-catenin signaling and VEGF up-regulation. Experimentally, cellular studies on DHT-induced aged DPCs confirmed PQFN’s antioxidant and antiaging mechanisms via oxidative stress and senescence assays. This represents the first application of gas-propelled microneedles for promoting bioactive delivering, confirming PQFN’s potential for effective AGA therapy. Similarly, Wang et al. [150] fabricated a curcumin–zinc framework (ZnMOF, Fig. 7A) encapsulated in a γ-polyglycolic acid microneedle patch (ZnMOF-MN) for transdermal delivery. In vitro studies showed that ZnMOF enhanced DPC viability and reversed DHT-induced inhibition. In vivo, ZnMOF-MN improved hair regrowth in AGA mice and enhanced angiogenesis.

Dissolvable microneedles are micron-scale needle arrays fabricated from biodegradable polymeric materials. Upon administration, they pierce the stratum corneum to create a microchannel. The encapsulated medication is then released straight into the skin tissues when the needle ends come into touch with the interstitial fluid in the subcutaneous layer, where they quickly break down. This technology offers the advantages of being painless and minimally invasive, enabling efficient transdermal delivery, while generating no sharp waste [151–153]. In a recent study, Xu’s team [154] developed a soluble microneedle patch loaded with glycosylated marine collagen peptides for hair loss treatment. Marine collagen peptides are rich in amino acids and possess antioxidant and anti-inflammatory capacities by reducing interleukin-1β (IL-1β) and tumor necrosis factor-α (TNF-α). To overcome peptides’ poor skin permeability, they employed HA-based dissolvable microneedles to encapsulate glycosylated marine collagen peptides. The microneedle array creates microchannels in the skin, facilitating direct peptide delivery to HFs while simultaneously inducing micro-injuries that stimulate vascularization, improve blood circulation, and promote growth factor production (Fig. 7B). This synergistic approach enhances collagen synthesis and fosters a favorable microenvironment for HFs, thus accelerating anagen phase’s transition and hair regrowth.

**Fig. 8.**
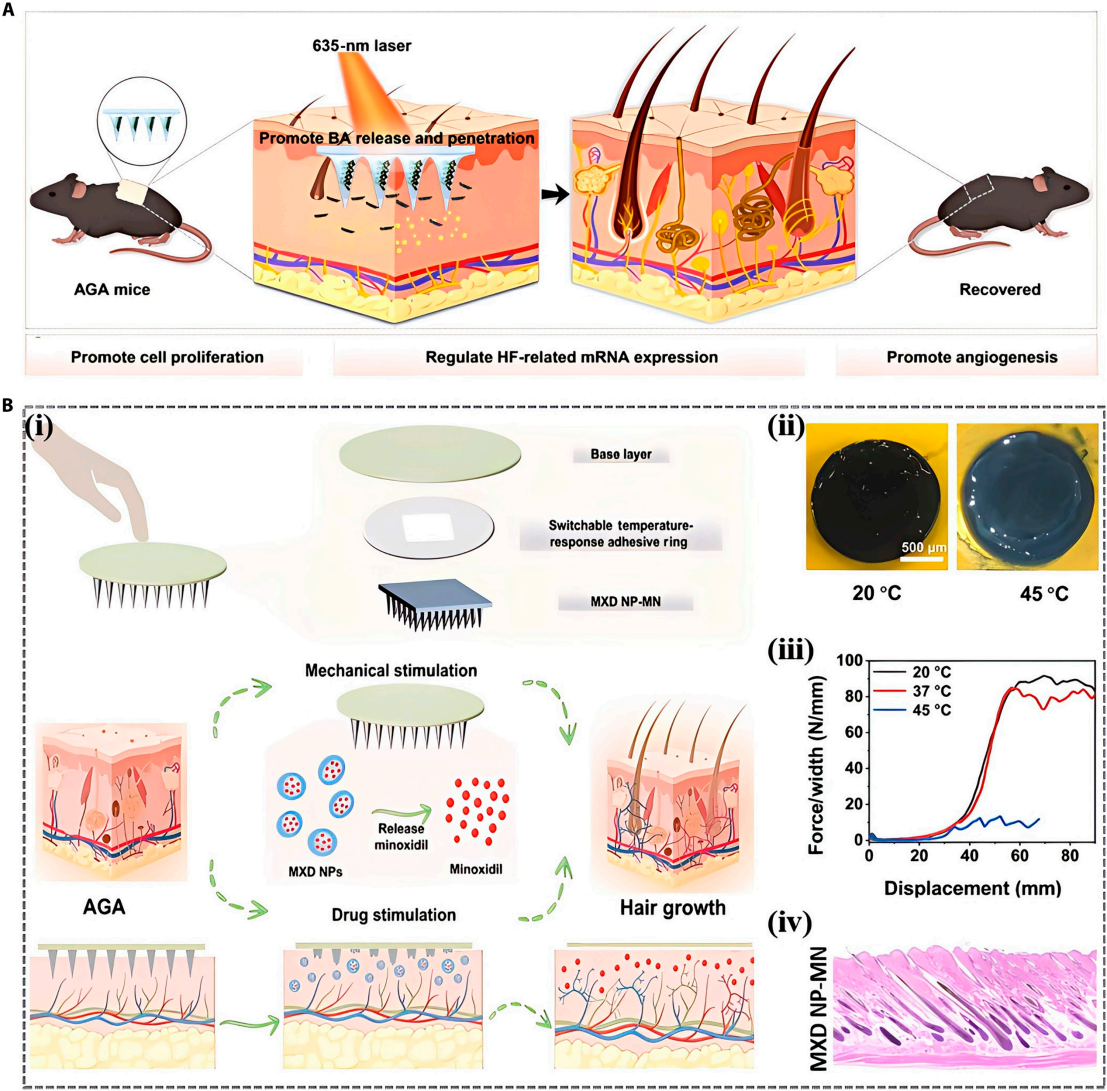
(A) Schematic illustration of BP-BA@MNs with 635-nm irradiation for anti-androgenetic alopecia (AGA) [158]. Copyright 2025, the author(s). (B) (i) Schematic illustration of the treatment of AGA through minoxidil nanoparticles-loaded microneedles. (ii) Digital photograph of the switchable temperature-responsive adhesion ring. (iii) Peeling test (180°) at different temperatures. (iv) In vivo assessment of hair regrowth [159]. Copyright 2024, Elsevier B.V.

More attractively, the creation of intelligent, responsive drug delivery systems that allow for on-demand and customized drug release control is greatly enhanced by the combination of microneedle materials with intelligent responsive elements [155–157]. Recently, for the treatment of AGA, a microneedle device containing black phosphorus nanosheets encasing baicalin (BP-BA MNs) was created, as depicted in Fig. 8A [158]. Here, upon 635-nm laser irradiation, BP-BA generated mild hyperthermia (about 42 °C), enabling controlled drug release and enhanced cellular uptake. This treatment modulated gene expression in DHT-treated human DPCs, down-regulating negative regulators (SRD5A2, AR, DKK1, and TGFB1) and up-regulating positive ones (CTNNBIP1, VEGFA). The microneedle with laser irradiation enhanced drug penetration and follicular accumulation. In vivo, this system achieved synergistic efficacy against AGA through combined chemotherapy, mild photothermal treatment, and microneedle delivery, demonstrating a satisfying safety profile. In a related research, Yuan’s group [159] combined a switchable, temperature-responsive adhesive ring with microneedles loaded with minoxidil nanoparticles (MXD NP-MN) (Fig. 8B). Minoxidil was encapsulated into nanoparticles using surfactants to improve its solubility and loaded into a HA microneedle array. The temperature-sensitive adhesion ring enabled stable skin attachment via thermal control and could be reversibly cleared to avoid secondary damage to HFs. In AGA mouse models, this system promoted the transition from telogen to anagen with lower dosage and reduced frequency, achieving superior therapeutic effects compared to repeated topical minoxidil solution application.

### Others

Platelet-rich plasma (PRP), an autologous plasma with platelet concentrations 3 to 5 times higher than whole blood, delivers growth factors (e.g., EGF, VEGF, IGF, FGF, and CCL2) that regulate HF cycling, such as stimulating anagen transition and recruiting macrophages to secrete regenerative factors. PRP subcutaneous injections are gaining popularity as an alternative to traditional AGA treatment because of its excellent outcomes, safety, and incredibly short recovery time [160]. In a study by Sultana and Paul, PRP injections demonstrated significantly greater efficacy than topical 5% minoxidil for treating AGA in 54 male participants over 12 weeks, though the contribution of the injection procedure itself remains unclear [161]. Expanding on this, Pakhomova and Smirnova [162] evaluated 69 males across 3 groups and found that while PRP monotherapy outperformed minoxidil alone, combination therapy (PRP plus topical minoxidil 5%) demonstrated better hair restoration results in comparison to either monotherapy, suggesting a synergistic effect. Despite the efficiency, no unified standards exist for its preparation, activation, or treatment regimens. Moreover, clinical injection would cause significant pain and patient discomfort.

As mentioned above, LLLT, particularly at 630 nm and 2 J/cm^2^, has shown promise by enhancing mitochondrial function, adenosine triphosphate production, and collagen synthesis without adverse effects. When combined with LLLT (630 nm, 2 J/cm^2^), active ingredients such as arginine, resveratrol, acetyl tetrapeptide-3, biotin tripeptide-1, artemia extract, and lindera strychnifolia root extract were assessed for their effects on collagen synthesis, fibroblast viability, mitochondrial activity, and proliferation. The most synergistic combination, termed CARRIPOWER, was further investigated in DPCs to elucidate mechanisms underlying hair loss treatment. Results demonstrated that this combination therapy reduced inflammatory cytokines and up-regulated growth factors, creating a pro-proliferative environment for HF cells. This innovative approach offers a promising new approach for treating AGA [163].

## Applications in AGA Treatment

With the deepening understanding of the pathogenesis of AGA, therapeutic strategies have evolved from broad-spectrum interventions toward precisely targeted regulation of specific pathological links. Drawing on an analysis of recent empirical studies, this section systematically elaborates on etiology-based targeted treatment strategies, the core of which lies in reshaping a healthy HF microenvironment by intervening in key pathological processes such as inflammation, oxidative stress, angiogenesis, and cell fate.

### Anti-inflammatory approaches

AGA is a progressive, genetically predisposed illness that is often marked by immunological dysregulation and inflammation [3,164,165]. Histologically, a cohort study of female patients with AGA demonstrated follicular miniaturization in nearly all biopsy specimens, with significant inflammatory infiltration observed in 86.2% of specimens, particularly pronounced in the infundibulum and isthmus regions [166]. At the molecular level, patients with AGA exhibit elevated inflammation- and apoptosis-related gene expressions like WNT7A, CASP7, and TNF [44]. Transcriptome analysis further confirms the up-regulation of proinflammatory regulators within affected HFs. This inflammatory state is exacerbated by androgens, which promote sebum production and generate proinflammatory cytokines, thereby accelerating disease progression. Critically, by secreting inflammatory cytokines, immune cells that infiltrate the follicular microenvironment, especially the bulge region that houses HFSCs, disturb HFSC homeostasis and interfere with the regular hair development cycle [68,167,168].

**Fig. 9.**
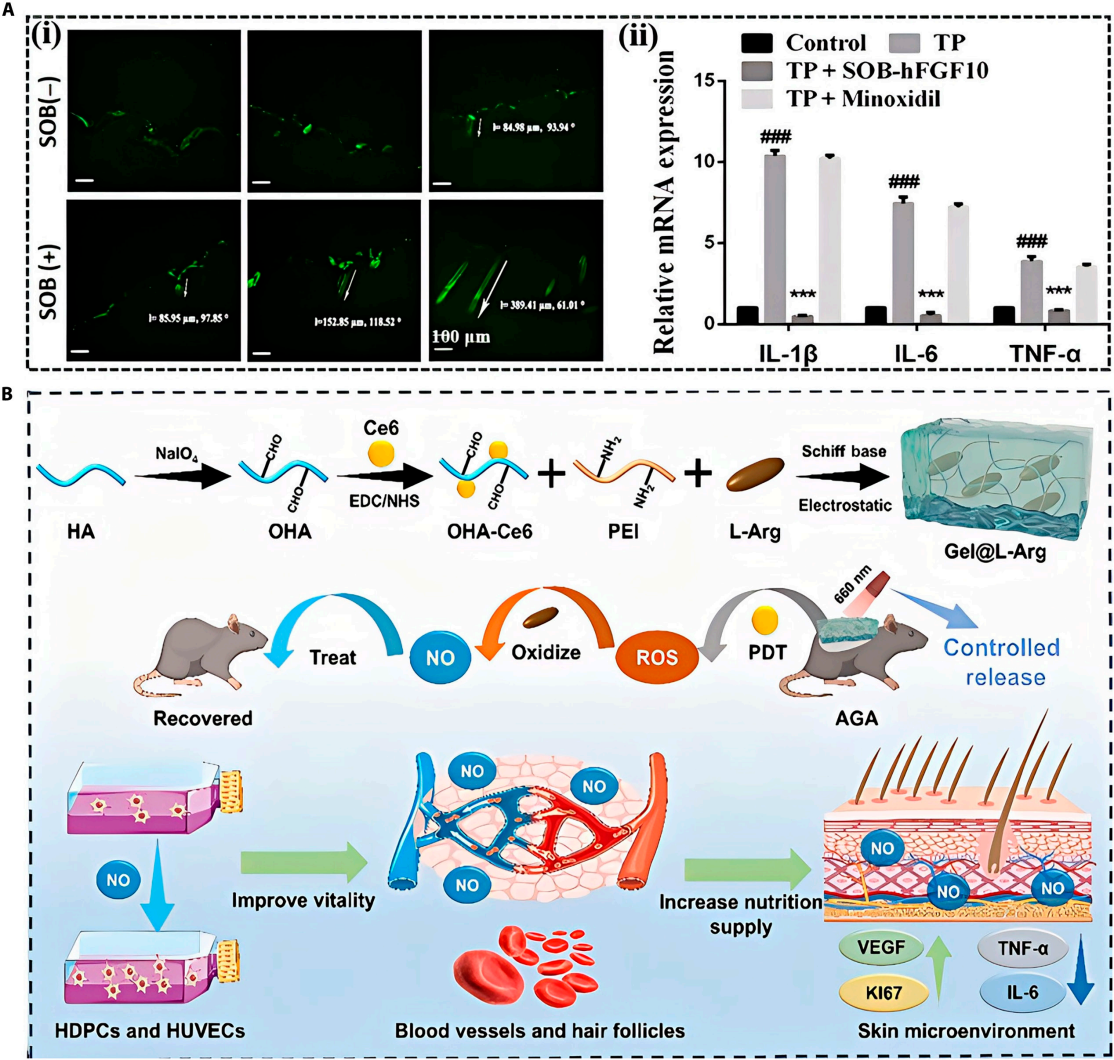
(A) (i) Permeable fluorescence microscopy images of mouse skin at various time points after topical application. (ii) mRNA levels of interleukin-1β (IL-1β), IL-6, and tumor necrosis factor-α (TNF-α) in mouse skin tissues. Here, TP means testosterone propionate. TP group compared with the control group, ###*P* < 0.001; TP + SOB-hFGF10 group and TP + Minoxidil groups compared with the TP group, ***P < 0.001 [169]. Copyright 2022, Elsevier B.V. (B) Schematic illustration of the near-infrared (NIR) light-triggered NO-releasing hyaluronic acid hydrogel transdermal delivery system for treating androgenetic alopecia (AGA). Here, HDPCs means human dermal papilla cells [170]. Copyright 2025, Elsevier B.V.

Given that, a human fibroblast growth factor 10-loaded safflower oil body (known as SOB-hFGF10) was created for the targeted AGA treatment, with a focus on investigating its immunomodulatory and anti-inflammatory mechanisms [169]. In terms of its anti-inflammatory properties, transcriptome sequencing showed that SOB-hFGF10 treatment down-regulated the expression of multiple proinflammatory genes and significantly enriched Gene Ontology terms associated with the immune system process, inflammatory response, and chemokine signaling pathways. In vitro, a coculture system of RAW264.7 macrophages and DPCs was established to simulate the microinflammatory environment of AGA. The results demonstrated that DHT stimulation significantly induced the expression and release of proinflammatory factors such as TNF-α, IL-1β, and IL-6 from macrophages (Fig. 9A). However, treatment with SOB-hFGF10 (100 ng/ml) for 24 h significantly reduced the levels of these inflammatory factors. By alleviating the inflammatory microenvironment, SOB-hFGF10 effectively restored the proliferative activity of DPCs and reduced DPCs’ total apoptosis rate from 8.59% in the DHT group to 5.93%. In vivo, SOB-hFGF10 significantly promoted hair regeneration, with hair coverage and length on day 24 approaching those of the healthy group. It also up-regulated the expression of follicle proliferation marker Ki67, the HF epithelial cell marker K5, and the DPC marker ALP. This study demonstrates that SOB-hFGF10, through targeted delivery of hFGF10 to HFs and regulating microinflammation, provides a potential method to treat AGA in the future.

In another study, a near-infrared (NIR) light-triggered nitric oxide (NO)-releasing HA hydrogel (Gel@L-Arg) was proposed and systematically evaluated for its therapeutic effects in AGA, as schemed in Fig. 9B [170]. In terms of immunomodulation and anti-inflammation, low-concentration NO dramatically decreased the mRNA and protein expression levels of IL-6 and TNF-α in damaged human DPCs and skin tissues, according to quantitative reverse transcription polymerase chain reaction and enzyme-linked immunosorbent assays. This suggests that NO has anti-inflammatory properties by preventing the release of inflammatory factors and enhancing the follicular microenvironment. The underlying mechanism is attributed to NO acting as a signaling molecule to regulate inflammation-related gene expression, thereby alleviating the immune response induced by DHT. Furthermore, upon NIR irradiation, the hydrogel generates ROS that catalyzes L-arginine (L-Arg) to release NO in a controlled and dose-tunable manner. Cell experiments demonstrated that low-concentration NO improved human umbilical vein endothelial cells’ and DPCs’ proliferation. It also up-regulated the expression of VEGF and CD31, thereby facilitating vascular repair. In animal studies, the Gel@L-Arg (0.5% + NIR) group exhibited a significant increase in HF count, vascular density, and expression of the proliferation marker Ki67, along with down-regulated AR expression, demonstrating notable efficacy in promoting hair regeneration.

### Antioxidant strategies

Oxidative stress is closely related with AGA. In AGA patients, elevated levels of oxidative stress are commonly observed around the HFs [3]. Physiological levels of ROS can modulate essential biological processes. However, the accumulation of excessive ROS leads to damage to key biomolecules such as DNA and proteins [171]. Oxidative stress brought on by elevated ROS levels inside and around HFs causes HFSCs to undergo apoptosis, which in turn inhibits hair growth [172]. In addition to being the main intracellular location for ROS synthesis, mitochondria are also important targets for damage caused by ROS. In order to preserve redox balance and energy metabolism homeostasis, cells often rely on mitophagy, a selective process that removes damaged mitochondria. Increased electron leakage and excessive ROS formation result from compromised mitochondrial function, such as decreased electron transport chain efficiency [3].

Facing this difficulty, Kong et al. [171] successfully constructed a HA-based dissolvable microneedle integrated with Cu_x_O nanozymes (Cu_x_O-MNs) for the treatment of AGA. First, Cu_x_O nanozymes with an average size of approximately 4.6 nm were synthesized via a facile and eco-friendly method, consisting of mixed phases of Cu^0^ and Cu₂O. In vitro antioxidant assays demonstrated that Cu_x_O nanozymes effectively scavenged multiple ROS, including hydroxyl radicals (•OH, with a scavenging rate of 87.6%), superoxide anions (•O₂^-^, with a scavenging rate of 90.4%), and 1,1-diphenyl-2-picrylhydrazyl (DPPH) radicals (with a scavenging rate of 76.6%). At the cellular level, pretreatment with Cu_x_O nanozymes significantly protected DPCs from H₂O₂-induced oxidative damage and restored the mitochondrial membrane potential decrease caused by carbonyl cyanide 3-chlorophenylhydrazone. After incorporation into HA microneedles, the resulting Cu_x_O-MNs were able to penetrate the skin and rapidly dissolve, leading to efficient delivery of Cu_x_O to perifollicular region. In vivo, Cu_x_O-MN treatment significantly reduced ROS levels around HFs (as validated by dihydroethidium staining and increased the expression of Ki67 and CD31, thus improving the transition from telogen to anagen. Following treatment, the Cu_x_O-MN group exhibited significantly superior hair coverage (74.0%), hair length (5.9 mm), and hair diameter (27.7 μm), achieving therapeutic effects comparable to minoxidil. Systematically, this study demonstrates that Cu_x_O-MNs effectively scavenge ROS, restore follicle microenvironment, and promote angiogenesis, indicating an efficient novel approach for future AGA therapy.

**Fig. 10.**
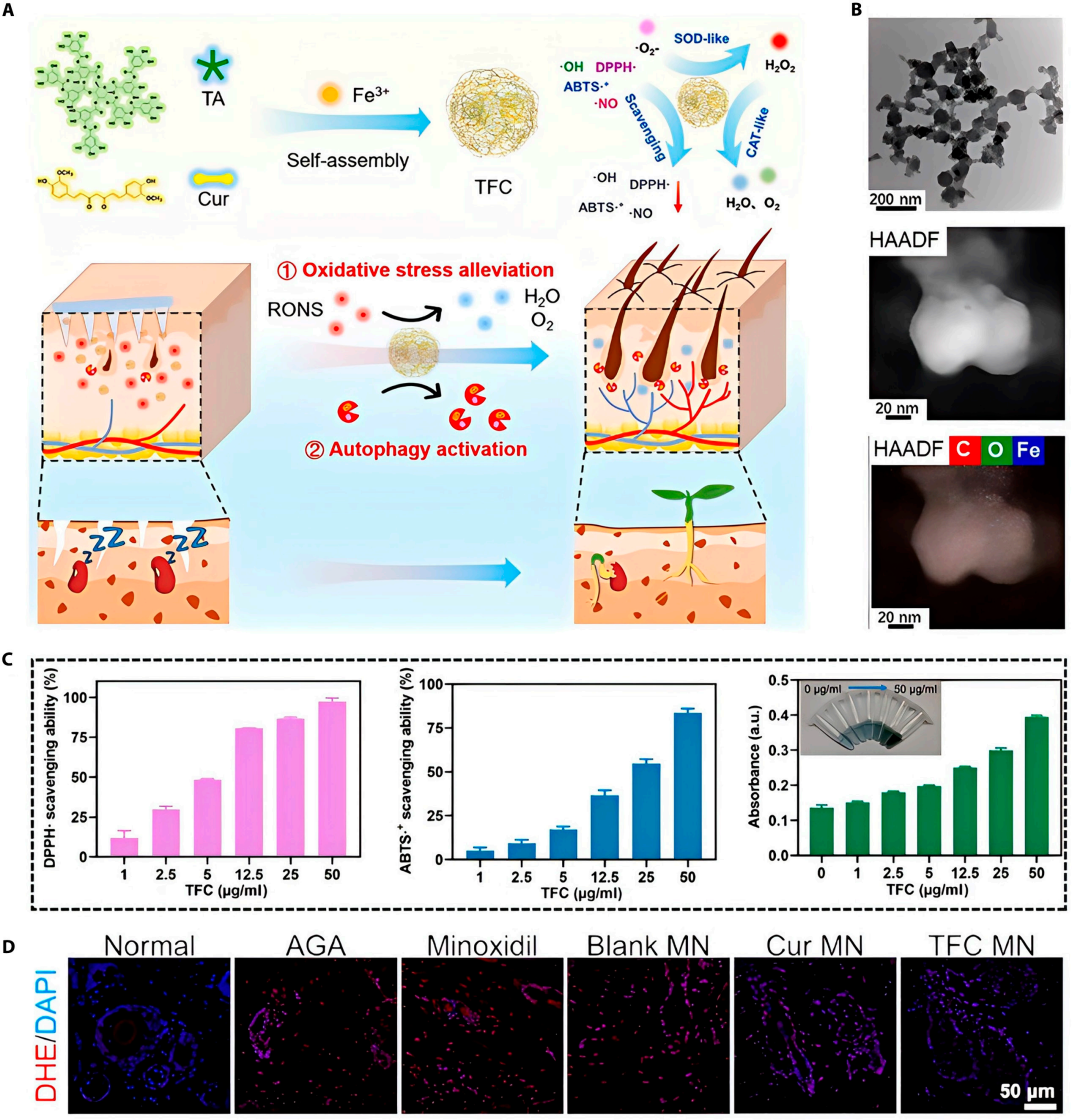
(A) Schematic diagram of the construction, mechanism, and application of TFC. (B) Transmission electron microscopy (TEM) image of TFC. (C) The clearance efficiency of Cu_x_O on different radicals. (D) Fluorescence microscopy images of reactive oxygen species (ROS) expression in skin tissue [173]. RONS, reactive oxygen and nitrogen species; HAADF, high-angle annular dark-field; DHE, dihydroethidium; DAPI, 4′,6-diamidino-2-phenylindole. Copyright 2025, the author(s).

In a related work, Chen et al. [173] proposed a curcumin (Cur)-based nanoformulation (TFC) grounded in the “seed-and-soil” theory for the targeted treatment of AGA (Fig. 10) [173]. TFC was fabricated via the self-assembly of tannic acid and Cur with Fe^3+^ through metal coordination, resulting in nanoparticles with a high drug loading capacity (52%) and excellent colloidal stability. Regarding the antioxidant mechanism, network pharmacology and molecular docking predicted that Cur regulates oxidative stress pathways. In vitro experiments confirmed that TFC possesses broad-spectrum ROS scavenging ability. Specifically, TFC (50 μg/ml) achieved scavenging rates of 97.42% and 83.51% against DPPH• and ABTS•^+^ radicals, respectively. Furthermore, leveraging the Fe^2+^/Fe^3+^ redox cycle, TFC mimicked superoxide dismutase (SOD) and catalase (CAT) activities, effectively scavenging •OH and •O₂^-^ and decomposing H₂O₂ to produce oxygen. At the cellular level, pretreatment with TFC (40 μM) significantly reduced intracellular levels of total ROS, •O₂^-^, •OH, and •NO in human DPCs induced by H₂O₂ and decreased the lipid peroxidation product malondialdehyde, maintaining cell viability above 95%. Upon incorporation into HA microneedles (TFC MN), the system exhibited enough strength (1 N/needle) for skin penetration and rapid dissolution. In an AGA mouse model, TFC MN treatment group was greatly superior to the model group. Dihydroethidium staining confirmed reduced ROS levels, while increased Ki67 and CD31 levels indicated enhanced cellular proliferation and angiogenesis. Up-regulation of LC3B confirmed the activation of autophagy.

### Angiogenesis promotion

Vascularization around HFs is another critically important for hair growth, as the start of anagen and the upkeep of the hair cycle are highly dependent on oxygen and nutrients supplied by the surrounding vascular network. According to existing research, keratinocytes in the outer root sheath increase the expression of VEGF during anagen, which causes strong perifollicular angiogenesis to satisfy the metabolic needs of the quickly growing HF. Conversely, these vessels regress during catagen and telogen [174]. However, in the balding scalp of patients with AGA, angiogenesis-related genes are significantly down-regulated, and the expression of VEGF, a key proangiogenic factor, is severely deficient. This directly restricts the nutrient supply to HFs and constitutes a critical pathological feature leading to follicle miniaturization and impaired hair regeneration [175]. Currently, restoring the perifollicular vascular network through therapeutic interventions has emerged as a promising research direction in AGA treatment. For instance, strategies such as minoxidil, direct VEGF supplementation, or the application of traditional Chinese medicine formulations and nanomedicines have been demonstrated to effectively promote perifollicular angiogenesis and improve the follicular microenvironment, thereby significantly increasing follicle size, accelerating hair regeneration, and enhancing hair quality [176,177].

**Fig. 11.**
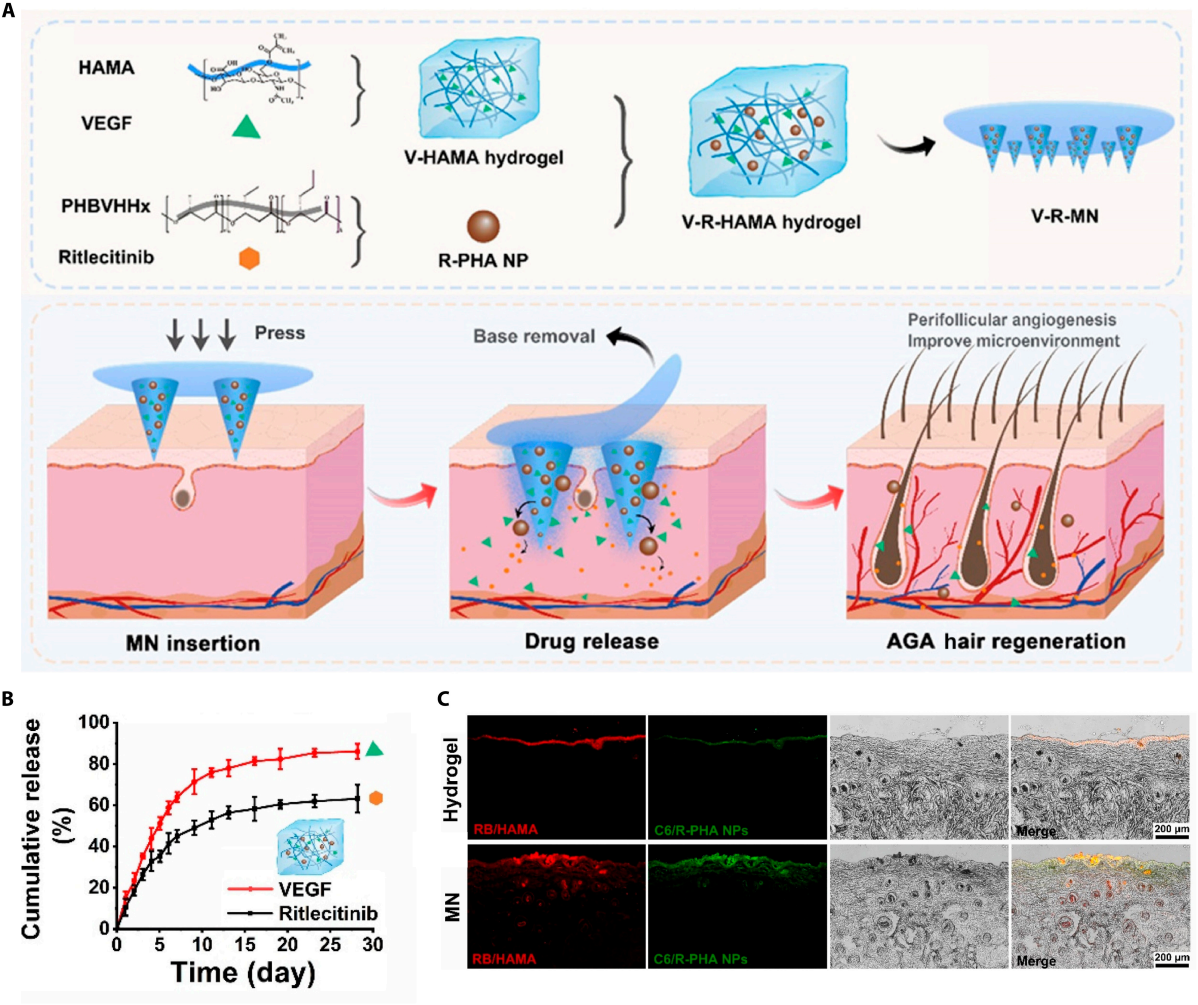
(A) Schematic illustration of the V-R-MN integrated microneedle patch for androgenetic alopecia (AGA) treatment. (B) Release of vascular endothelial growth factor (VEGF) and ritlecitinib from V-R-HAMA. (C) Representative fluorescence images of skin sections 24 h after topical application [175]. Copyright 2024, the author(s).

**Fig. 12.**
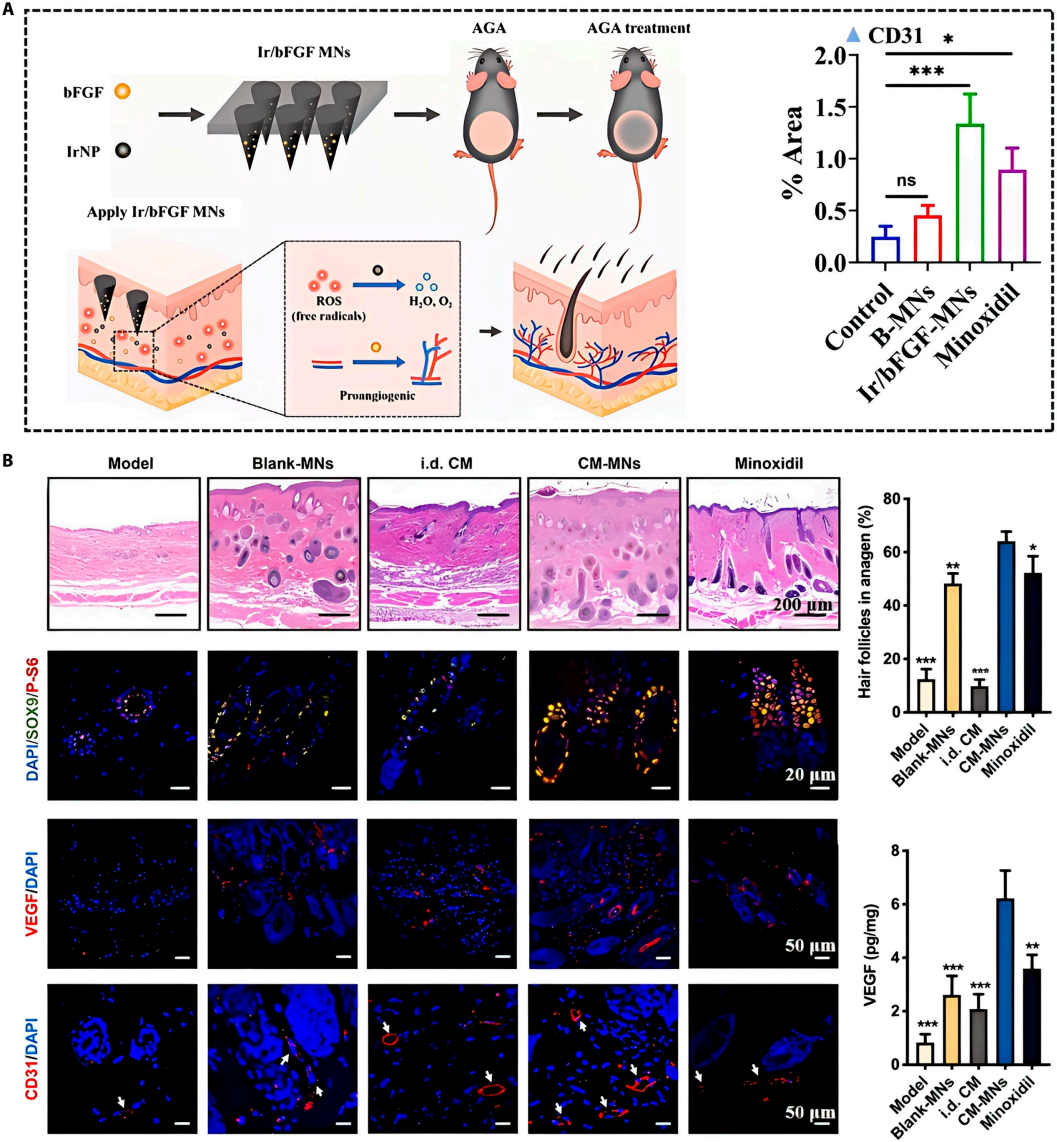
(A) (i) Schematic diagram of water gel microneedles containing basic fibroblast growth factor (bFGF) and polyvinyl pyrrolidone (PVP)-modified iridium nanoparticles for androgenetic alopecia (AGA) treatment. (ii) Quantitative analysis of the CD31 expression [178]. Copyright 2025, Elsevier B.V. (B) Evaluation of hair regrowth in an AGA mouse model. Here, i.d. CM is the intradermal injection of conditioned medium [179] Copyright 2022, Elsevier B.V.

In the aspect of delivering growth factors, a HA microneedle patch coloaded with VEGF and ritlecitinib (V-R-MNs) was recently created for the minimally invasive AGA therapy (Fig. 11) [175]. Regarding angiogenesis promotion, perifollicular vascular density was evaluated by CD31 immunohistochemical staining. The results demonstrated that the V-R-MN group exhibited the best condition of blood vessels, significantly outperforming the minoxidil group. This confirms that the released VEGF effectively promotes perifollicular angiogenesis. The underlying mechanism is attributed to VEGF, as a key proangiogenic factor, directly inducing vascular endothelial cells’ proliferation, thereby improving the nutrient supply to HFs. Moreover, the inherent mechanical stimulation also contributes to proangiogenic effects. Ritlecitinib improves the perifollicular immune microenvironment by inhibiting the Janus kinase 3 signaling pathway. The microneedle system also exhibits excellent mechanical strength and biosafety, enabling sustained and prolonged drug release. By synergistically promoting angiogenesis, modulating the microenvironment, and providing mechanical stimulation, V-R-MNs significantly promoted hair regrowth in vivo, demonstrating superior efficacy to the clinical drug minoxidil. Besides, Ding et al. [178] developed GelMA hydrogel microneedles loaded with basic fibroblast growth factor (bFGF) and iridium nanoparticles, named as Ir/bFGF MNs, for the treatment of AGA (Fig. 12A). In vitro tube formation assays confirmed that the microneedle promoted the formation of tubular structures in human umbilical vein endothelial cells, while enzyme-linked immunosorbent assay and Western blot analyses revealed up-regulated expression of VEGF and VEGF-R. As for the system’s underlying mechanism, bFGF could directly induce vascular endothelial cells’ activity. Meanwhile, the mechanical stimulation generated by microneedle penetration also helps vessel regeneration. In vivo, CD31 immunohistochemical staining demonstrated an obvious increase in perifollicular vascular density in bFGF-involved group, together with superior hair growth coverage, HF count, and hair quality.

**Fig. 13.**
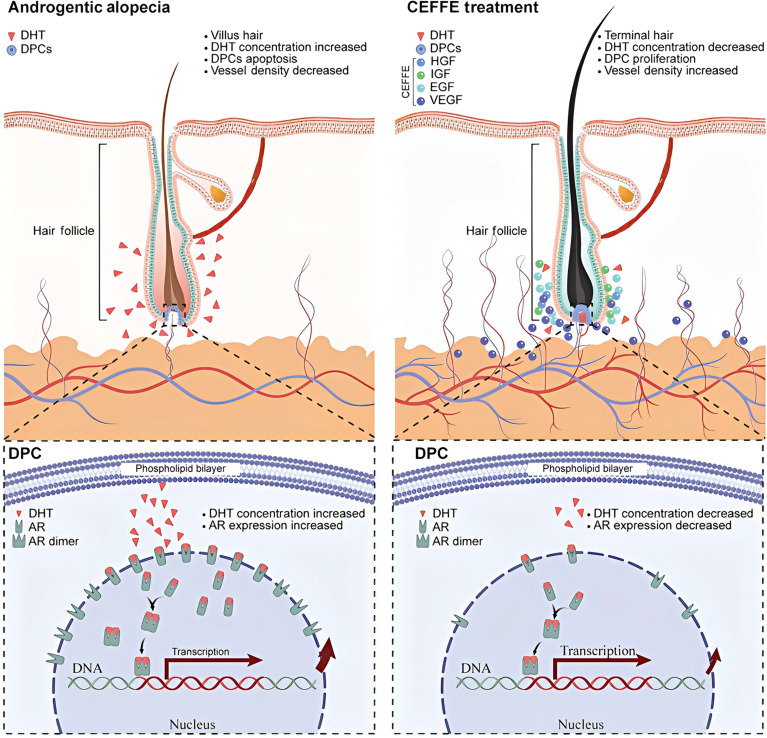
Cell-free fat extract (CEFFE) promotes hair growth in androgenetic alopecia (AGA) via mediating dihydrotestosterone (DHT)/AR pathway in dermal papilla cell (DPC) to enhance cell proliferation, modulate the cell cycle, and increase neovascularization in the DHT-induced model [180]. Copyright 2023, the author(s).

In addition, dissolvable microneedle patches encapsulated with conditioned medium derived from hypoxia-preconditioned mesenchymal stem cells (CM-MNs) have been proposed for curing AGA (Fig. 12B) [179]. Regarding angiogenesis promotion, hypoxic preconditioning enriched the CM with proangiogenic factors (hypoxia-inducible factor 1-α [HIF-1α]), which synergized with the mechanical stimulation generated by microneedle penetration to collectively remodel the perifollicular microvasculature. In vivo results demonstrated that CM-MNs significantly increased perifollicular vascular density, thereby activating HFSCs and promoting hair regrowth. Relatively, Zhang’s team [180] evaluated the efficacy and underlying mechanism of cell-free fat extract (CEFFE) in AGA management, as demonstrated in Fig. 13. Regarding angiogenesis promotion, in vivo experiments confirmed through CD31 staining that CEFFE significantly increased perifollicular capillary density, while synergistically promoting the proliferation of DPCs. CEFFE also antagonized DHT-induced apoptosis by down-regulating AR expression and reducing intracellular DHT levels. Animal studies demonstrated that the CEFFE-treated groups exhibited significantly enhanced hair growth rate and coverage, along with increased HF counts, indicating that CEFFE effectively treats AGA through the dual mechanisms of promoting angiogenesis and regulating the DHT/AR signaling pathway.

### Cell fate modulation

**Fig. 14.**
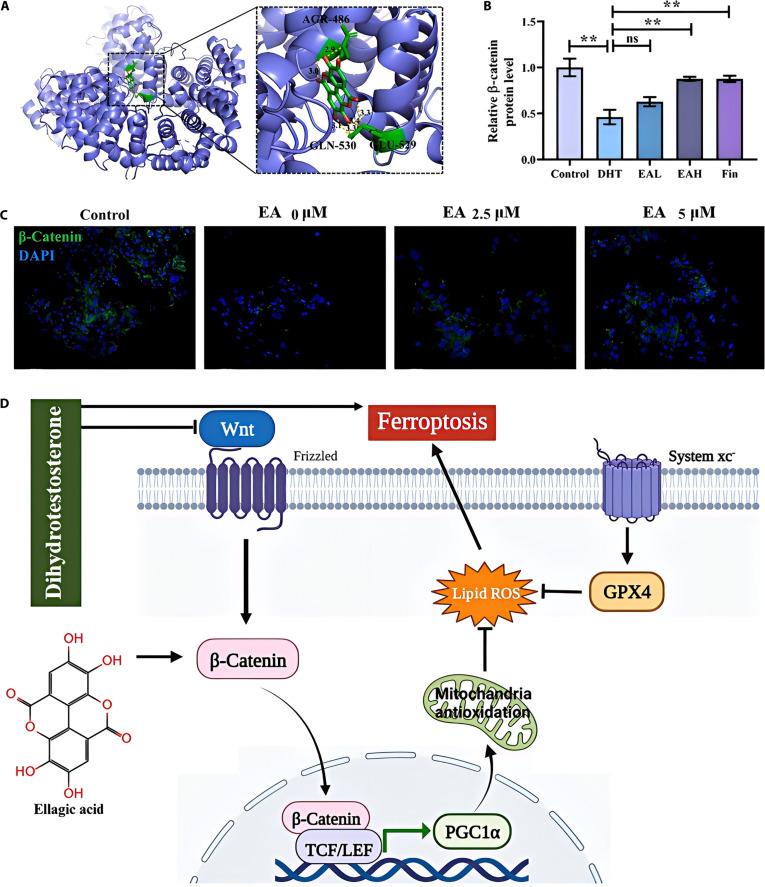
(A) Molecular docking of ellagic acid (EA) with β-catenin. (B) Promotion of dihydrotestosterone (DHT)-inhibited β-catenin expression by EA in skin tissues. Here, EAL is ellagic acid-low, EAH is ellagic acid-high, and Fin is finasteride. (C) Stimulation of DHT-attenuated nuclear transport of β-catenin in dermal papilla cells (DPCs) by EA. (D) Mediation of alleviating DHT-induced ferroptosis [181]. DAPI, 4′,6-diamidino-2-phenylindole. Copyright 2024, Elsevier B.V.

Fu et al. investigated the mechanism by which ellagic acid (EA) promotes hair regeneration through regulating cell fate, specifically by inhibiting DHT-induced ferroptosis in DPCs (Fig. 14) [181]. Regarding cell fate regulation, the study first confirmed that DHT induces ferroptosis in DPCs via detecting iron content, lipid peroxidation (malondialdehyde, 4-hydroxynonenal), glutathione levels, and the expression of GPX4 and SLC7A11. EA treatment significantly reversed these indicators and alleviated cellular damage. Transmission electron microscopy revealed that EA ameliorated DHT-induced mitochondrial shrinkage and cristae loss, demonstrating its protective effect on mitochondrial function. Mechanistic studies showed that EA regulates cell fate through activating the Wnt/β-catenin signaling pathway. Western blot and immunofluorescence analyses further demonstrated that EA up-regulated the expression of Wnt5a and β-catenin and promoted their nuclear translocation. Molecular docking further confirmed the binding potential between EA and β-catenin. Following β-catenin knockdown via siRNA, the inhibitory effects of EA on DHT-induced lipid peroxidation, GPX4 down-regulation, and mitochondrial ROS accumulation were significantly attenuated, confirming β-catenin as a key target of EA in regulating ferroptosis. Additionally, EA restored DHT-inhibited PGC-1α expression, thereby improving mitochondrial biogenesis. In vivo experiments demonstrated that oral administration of EA dose-dependently promoted hair growth in an AGA mouse model, increased HF count, and reduced DHT levels and AR expression in both serum and skin tissue. In conclusion, EA effectively treats AGA by activating the Wnt/β-catenin pathway and inhibiting DHT-induced ferroptosis in DPCs to reshape cell survival fate.

### Synergistic therapy

**Fig. 15.**
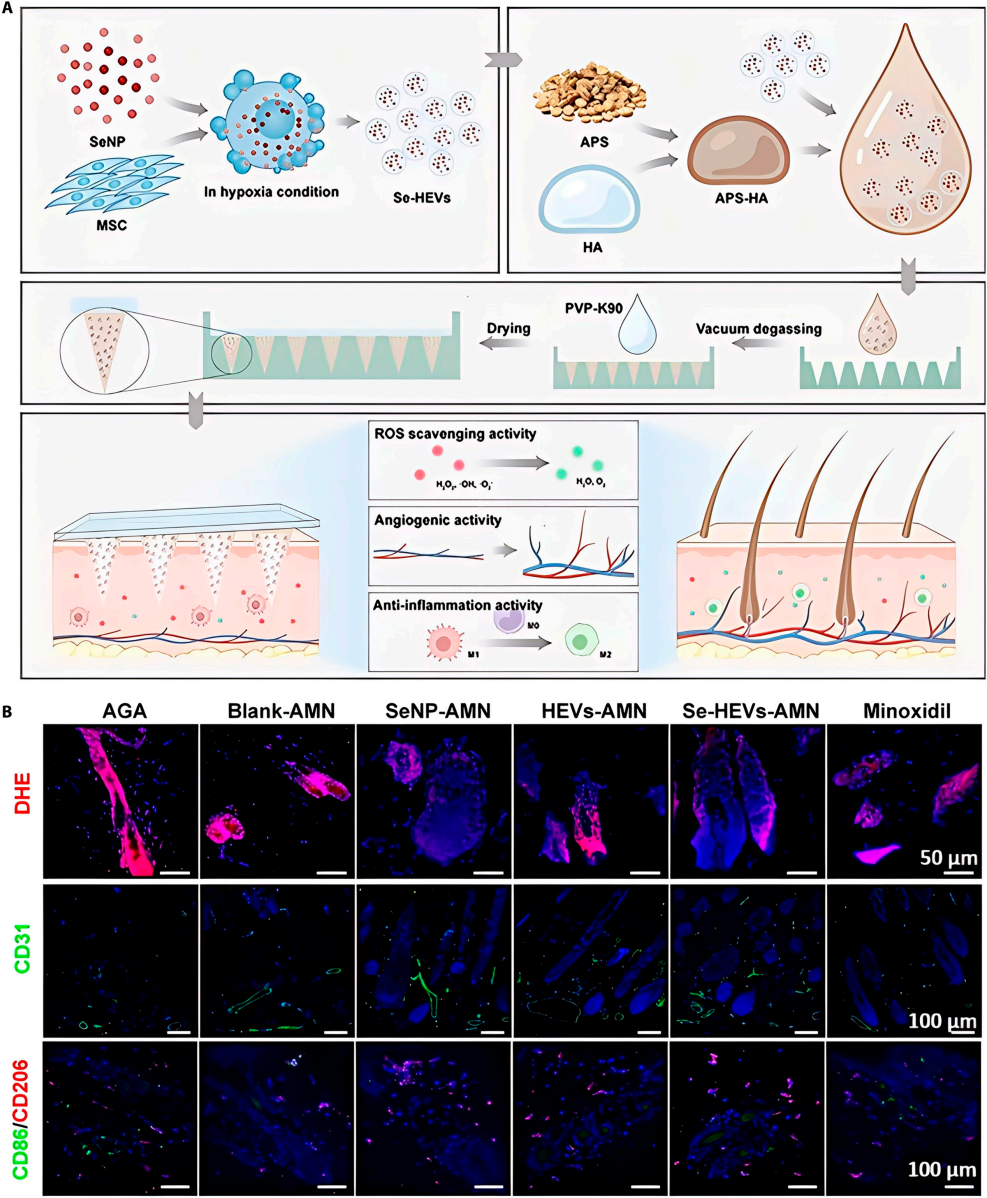
(A) Schematic illustration of an astragalus polysaccharide microneedle (AMN) comprising hypoxic extracellular vesicle (EV)-encapsulated SeNPs (Se-HEVs-AMN) patch for androgenetic alopecia (AGA). (B) Se-HEVs-AMN promotes hair growth by improving the microenvironment [182]. Copyright 2025, the author(s).

To establish a favorable perifollicular microenvironment, current therapeutic strategies for AGA have evolved from single-drug approaches toward multimodal synergistic intervention. By simultaneously targeting multiple pathogenic factors—including oxidative stress alleviation, angiogenesis promotion, inflammation inhibition, and hormonal regulation—these multimodal therapies achieve systemic remodeling of the HF microenvironment and have become the mainstream trend in clinical practice. In a typical study, a synergistic therapeutic system based on selenium nanozymes, hypoxic exosomes, and astragalus polysaccharide microneedles was developed for the treatment of AGA, as schemed in Fig. 15A [182]. Experiments demonstrated that selenium nanozymes scavenged ROS through their antioxidant enzyme-like activities, while hypoxic exosomes carried HIF-1α to up-regulate VEGF expression, thereby improving perifollicular blood supply. Additionally, astragalus polysaccharide microneedles loaded with anti-inflammatory components inhibited M1 polarization of macrophages. In vitro experiments confirmed the synergistic effects of this system in exerting antioxidant, proangiogenic, and anti-inflammatory functions (Fig. 15B). In animal models, the combination treatment group exhibited significantly superior outcomes in HF count, vascular density, and anagen phase initiation compared to monotherapy groups, demonstrating the efficacy of multitarget synergistic remodeling of the HF microenvironment.

**Fig. 16.**
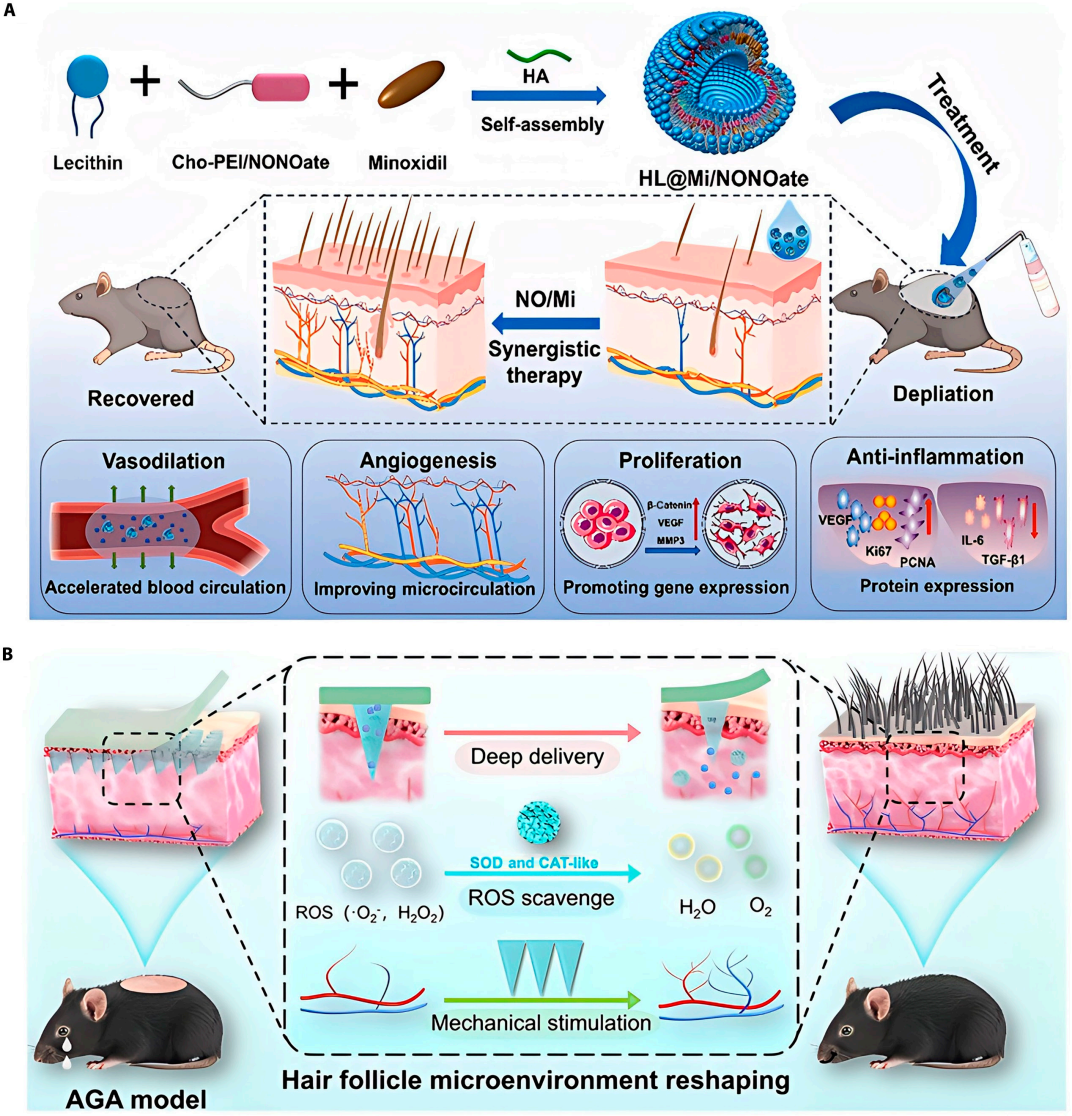
(A) Schematic diagram of HL@Mi/NONOate transdermal delivery platform design route and synergistic treatment of androgenetic alopecia (AGA) [183]. Copyright 2025, the author(s). (B) Schematic illustration of the application of NC&minoxidil microneedles [184]. Copyright 2025, American Chemical Society.

Ma’s team [183] proposed a synergistic therapeutic system (HL@Mi/NONOate) based on a HA liposome composite carrier (HL) for delivering minoxidil and NO, as depicted in Fig. 16A. The system was fabricated via self-assembly of lecithin and HA with a NO donor derived from small-molecular weight polyethyleneimine-modified cholesteryl chloroformate, which encapsulated Mi within the liposomes. In terms of synergistic therapy, NO activated guanylate cyclase to elevate cyclic guanosine monophosphate levels, inducing local vasodilation and increasing blood flow, thereby significantly enhancing the skin penetration and retention of Mi. Concurrently, NO itself promoted cell proliferation, migration, and angiogenesis (via up-regulation of VEGF and HIF-1α), while exerting anti-inflammatory effects. In vitro experiments confirmed that HL@Mi/NONOate enhanced DPC viability and promoted tube formation in vascular endothelial cells. In an AGA mouse model, the combination treatment group exhibited significantly superior outcomes in HF count, vascular density, and anagen phase initiation compared to the minoxidil monotherapy group, achieving synergistic hair growth promotion through modulation of cell differentiation pathways. This multitarget system offers an efficient and safe therapy for AGA through dual mechanisms of NO-mediated permeation enhancement and synergistic treatment.

Moreover, dissolvable HA microneedle systems coloaded with Ni-Cu (NC) nanozymes and minoxidil (NC&minoxidil MNs) were constructed for the synergistic treatment of AGA (Fig. 16B) [184]. Regarding experimental design, NC nanozymes, leveraging the mixed valence states of Ni and Cu, exhibited both SOD-like and CAT-like activities, synergistically eliminating excessive superoxide anions and hydrogen peroxide in the HF microenvironment, thereby effectively alleviating oxidative stress. The microneedle system, fabricated with low-molecular weight HA as the tip material, successfully penetrated the skin barrier after loading NC and minoxidil, achieving precise delivery of both agents to the perifollicular region. The polyvinylpyrrolidone backing layer ensured adequate mechanical strength of the microneedles and facilitated rapid separation of the tips from the backing postinsertion. In terms of synergistic therapy, NC scavenged ROS to protect DPCs from oxidative damage, while minoxidil delivered via microneedles overcame the limitation of low transdermal absorption associated with conventional administration. Concurrently, the mechanical stimulation generated by microneedle puncture promoted local angiogenesis and improved blood perfusion. In vitro experiments confirmed that NC possessed efficient SOD/CAT mimetic enzyme activities, and cellular assays demonstrated that NC&minoxidil MNs significantly enhanced DPC viability while inhibiting apoptosis. In an AGA mouse model, the combination treatment group exhibited significantly superior outcomes in HF count, vascular density, and anagen phase hair coverage compared to monotherapy groups, achieving multitarget synergistic effects encompassing antioxidant activity, angiogenesis promotion, and enhanced drug efficacy. This study provides a safe and efficient therapeutic strategy for AGA.

## Summary and Outlook

AGA, as a typical form of alopecia disorders, is characterized by complex pathological mechanisms involving genetic susceptibility, androgen metabolism dysregulation, and perifollicular microenvironment imbalance. Current clinical interventions, including minoxidil, finasteride, laser therapy, and hair transplantation, can alleviate symptoms to some extent. However, they are generally limited by suboptimal efficacy, notable adverse effects, and inability to halt disease progression. Up to date, the rapid advancements of biomedical technologies have ushered in new opportunities for AGA treatment. This review systematically summarized the AGA’s pathogenesis and existing clinical therapies’ applications and limitations, with a particular focus on the progress of cutting-edge biomedical technologies in AGA treatment, including novel pharmacological formulations, nanotechnology, stem cell-based therapies, and advanced microneedle platforms. Furthermore, from the perspective of core pathogenic factors such as inflammation, oxidative stress, angiogenesis, and combination therapy, representative case studies have been used to analyze the design principles and underlying mechanisms of the current therapeutic strategies.

Despite the tremendous potential demonstrated by biomedical technologies in the field of AGA treatment, related research remains in its early stages, and numerous challenges urgently require resolution. First, most novel drug delivery systems, such as nanocarriers and microneedles, are still confined to animal studies, and their safety, stability, and scalability for mass production have yet to be fully validated, posing significant obstacles to clinical translation. Second, regarding stem cell- and exosome-based therapies, although they possess promise in hair regenerative field, their mechanisms of action are complex, and the absence of standardized protocols for cell sources, culture conditions, extraction, and purification leads to substantial heterogeneity in therapeutic outcomes, hindering precise regulation. Additionally, existing studies predominantly focus on interventions targeting single molecules or pathways; however, as a multifactorial disorder, AGA often cannot be adequately managed by blocking a single pathological node. More importantly, there remains a lack of in-depth investigation into the synergistic mechanisms underlying combination therapeutic strategies, with critical issues such as the interactions between different treatment modalities, administration timing, and dosage ratios remaining poorly understood, thereby limiting their clinical applicability.

To sum up, future research directions for AGA treatment could focus on the following aspects. First, to achieve spatiotemporally precise control over drug release and maximize therapeutic efficacy while reducing side effects, intelligent and personalized drug delivery systems (like combining microneedles with stimuli-responsive materials) are crucial. Furthermore, the advancement of sophisticated materials capable of monitoring the microenvironment and providing feedback regulation would facilitate on-demand drug release during the treatment process. Second, in-depth exploration of the mechanisms underlying stem cell- and exosome-based therapies, along with the establishment of standardized preparation and quality control systems, is imperative to promote their clinical translation. Third, greater emphasis should be placed on investigating multitarget combination therapeutic strategies, employing systems biology and network pharmacology approaches to elucidate the interactions between different pathogenic pathways and construct synergistic treatment regimens. For example, a topical formulation (clascoterone) featuring multitarget antagonism of the AR and prostaglandin pathways is currently undergoing Phase II/III trials. Finally, efforts to bridge basic research and clinical needs should be intensified, including the development of animal models that more accurately recapitulate human pathological features, and the promotion of high-quality, multicenter clinical trials, with the ultimate goal of providing safer, more effective, and durable therapeutic options for AGA patients.
